# Nuclear Quantum Effects on the Nature of Hydroboration Selectivity: Experimental Effects of First-Collision Tunneling

**DOI:** 10.1021/jacs.4c09306

**Published:** 2024-09-16

**Authors:** Christoph E. Bracher, Connor J. Allen, Daniel A. Singleton

**Affiliations:** Department of Chemistry, Texas A&M University, P.O. Box 30012, College Station, Texas 77842, United States

## Abstract

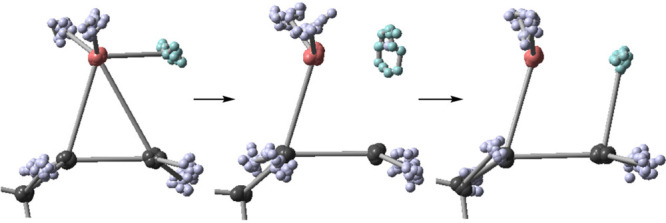

The understanding of selectivity in reactions exhibiting nonstatistical dynamics is impeded by the limitations of trajectory studies with regard to nuclear quantum effects, especially tunneling. We described here the use of ring-polymer molecular dynamics (RPMD) to account for an unusual regiochemical isotope effect on the regioselectivity of hydroborations of alkenes with BH_3_/BD_3_. RPMD is able to account for the experimental observation, while statistical approaches and classical trajectories fail. The combination of experiment and RPMD trajectories suggests that tunneling in the initial collision of reactants is the major source of the nonstatistical selectivity.

The understanding of reaction selectivity is central to the design and control of reactions. We describe here computational and experimental support for a previously unrecognized factor in selectivity, first-collision tunneling,^1^ and how this impacts the regioselectivity of hydroborations with BH_3_.

Kinetic selectivity is usually understood within the context of statistical rate theories such as transition state theory (TST). However, such theories can fail in the prediction of experimental observations under a variety of circumstances.^2−5^ When this happens, theory can fall back on the use of dynamical trajectories to predict observations. The idea is that trajectories are in a sense primordial; they avoid potentially inapplicable assumptions such as the statistical distribution of vibrational energy, as well as approximations such as that of harmonicity. Trajectory studies have been able to successfully predict many observations where statistical theories fail or are inapplicable.^2−5^

Trajectory studies themselves, however, usually involve severe unphysical approximations. Fully classical trajectories ignore the quantum nature of nuclei and propagate atomic motions with no regard to the quantization of vibrational modes, zero-point energy (ZPE), or tunneling. Quasiclassical trajectories (QCTs) and related methods can mimic ZPE effects, but they introduce the additional problem of energy leakage from normal modes. QCTs are then manifestly inapplicable to longer simulations and at times subject to extreme errors even on short time scales.^6^ The reaction studied here is pernicious for both QCTs and classical trajectories due to the expected importance of tunneling and ZPE in a hydrogen-transfer process and a necessarily long simulation time. Exact quantum dynamics employing wave function methods would be the ultimate solution to these problems, but they are impractical for systems involving more than a few atoms.

Ring-polymer molecular dynamics (RPMD) provides an approximation of quantum trajectories based on Feynman’s path-integral formulation of quantum mechanics.^7−9^ RPMD is a model, with established theoretical inaccuracies and artifacts,^10−12^ but it is computationally efficient and remarkably successful in the emulation of tunneling and ZPE effects in diverse systems.^13−18^ RPMD has been used for nonequilibrium dynamics,^19,20^ but it was not designed for them. RPMD’s application here to the complex nonequilibrium hydroboration reaction requires approximations of unknown impact. Our approach then is experimental. We find that RPMD accounts for an experimental observation that is not predictable by alternative theoretical methods. The critical stumbling block is tunneling in the initial collision of BH_3_ with alkenes, which we find is the major source of the nonstatistical selectivity that we have previously observed.

The hydroboration of terminal alkenes (**1**) with BH_3_ affords a mixture of regioisomeric “anti-Markovnikov” (**anti-Mark**) and “Markovnikov” (**Mark**) products (Figure 1). TST and canonical variational TST (CVT)calculations including small-curvature tunneling (SCT) predict that only 1–3% of the product should be Markovnikov.^4^ In contrast, we have previously observed that 9–11% of **Mark** is formed experimentally.^4^

**Figure 1:**
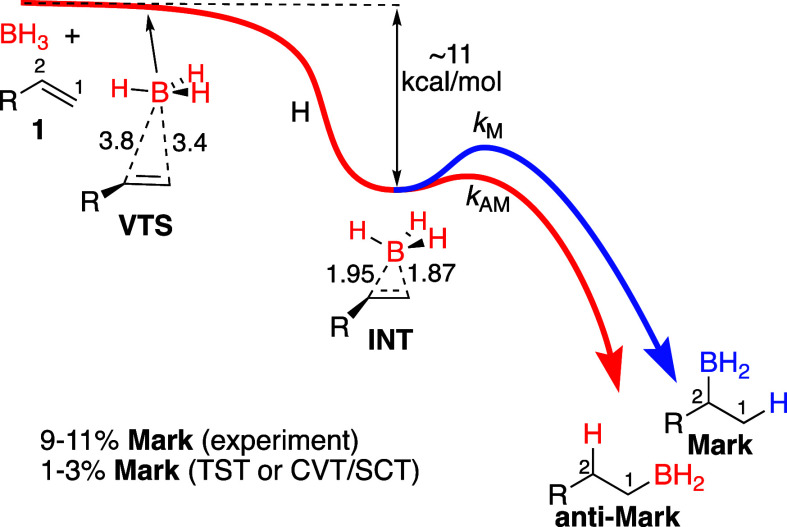
Energy profile and selectivity in the hydroboration of alkenes.

In general terms, the discrepancy between experiment and TST or CVT/SCT predictions for hydroboration arises from the excess energy generated in the enthalpically barrierless approach of BH_3_ to an alkene, forming a “hot” π complex **INT**. The nonstatistical nature of this reaction is supported by multiple lines of independent evidence.^4^ It had been proposed that the selectivity could be explained using either statistical approaches allowing for the excess energy, e.g. RRKM-master equation (RRKM-ME) calculations, or phase-space approaches.^21^ However, RRKM-ME and other statistical and nonstatistical rate models failed to account for alkyl chain-length effects on the selectivity, at best approaching consistency with experiment using an *ad hoc* parametrized model.^4b^

The hydroboration of 1-hexene (**1**, R = *n*-butyl) with a large excess of BH_3_ at 25 °C affords a mixture of the **anti-Mark** and **Mark** regioisomers with 10.7 ± 0.5% **Mark** (Figure 2).^4b^ Under identical conditions, BD_3_ affords only 6.1 ± 0.4% **Mark** (from seven measurements on independent reactions, uncertainties are 95% confidence limits). This regiochemical kinetic isotope effect (KIE) of 1.8 ± 0.2 (defined as (*k*_M_/*k*_AM_)_H_/(*k*_M_/*k*_AM_)_D_) is unusual, arising as it does from the competition between closely analogous reactions. More importantly, the KIE is unpredictable from present chemical theory. The large errors in the predicted KIE from the statistical CVT/SCT and RRKM-ME methods (Figure 2) are not surprising, given their prior failure to account for the simple **anti-Mark**/**Mark** product ratio.^4b^ Classical trajectories initialized from the area of the canonical variational transition state (**VTS**) do account crudely for the BH_3_ product ratio (predicting 9.8% **Mark**), as we have seen previously,^4^ but they naturally fail on the KIE due to the absence of quantum effects.

**Figure 2:**
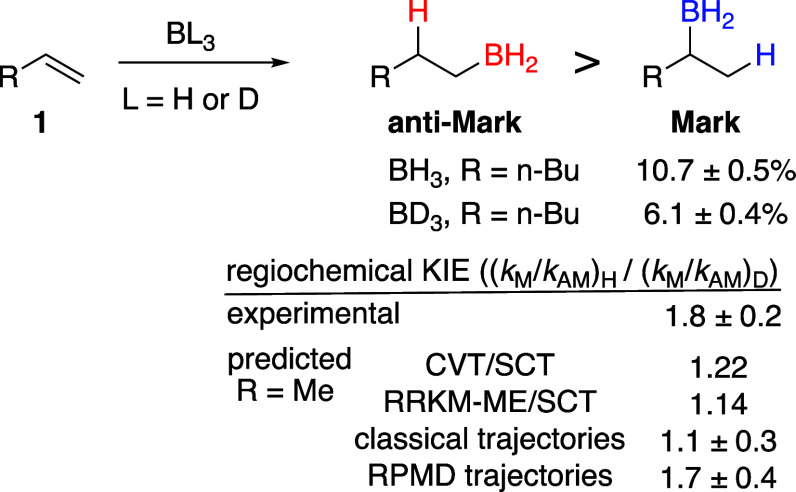
Experimental and predicted selectivities and regiochemical KIE for hydroborations. Predicted KIEs are for R = methyl. See the Supporting Information for calculational details.

The application of RPMD trajectories to this nonequilibrium reaction faces the problem of how to approximate the temperature of the internal ring-polymer modes.^19b^ As a minimalist approach, trajectories were simply thermostated at 25 °C while constrained in the area of **VTS**, then snapshots were extracted and propagated forward and backward in time, without constraint or further intervention, until either the **anti-Mark** or **Mark** products were formed or the reactants separated. The trajectories employed a modified version of the available RPMDrate code,^22^ including here the use of on-the-fly DFT^23^ force calculations from Gaussian 16^24^ with efficient parallelization (see the Supporting Information (SI) for the code, trajectory details, and an examination of real/imaginary energy conservation). The RPMD trajectories were carried out using from 1 to 32 beads and both propene and 1-hexene in exploratory studies, with the bulk production trajectories using 16 beads and propene. To gauge trajectory validity, we first explored 16-bead RPMD trajectories started from the area of the π-complex **INT**, that is, with no extra energy. These afforded 2.1% **Mark** (142:3), which fits closely with the CVT/SCT statistical prediction (2.5%), but this is of course not consistent with experiment.

When appropriately initialized from the area of **VTS**,^25^ however, the **Mark**-forming trajectories employing BH_3_ increased dramatically, to 15.3% (56 out of 366 product-forming trajectories). Using BD_3_, the amount of **Mark** product was only 9.5% (32 out of 336). These results lead to a predicted^26^ KIE of 1.7 ± 0.4, which is indistinguishable from the experimental KIE. This correspondence suggests that the RPMD trajectories are reasonably approximating the combination of nonstatistical dynamics and tunneling that the statistical methods and classical trajectories cannot.

Closer examination of the RPMD trajectories reveals a series of exceptional features. The most striking of these is that ∼70% of the **Mark** product is formed *in the initial collision of the BH*_3_*with propene* (defined as the first time a C–B distance is within 2.0 Å). This is more than double what is seen in either the classical or BD_3_ trajectories. The importance of the collisional reactions can be seen in the precipitous drop in the Markovnikov trajectory survivor profile in the first 500 fs (Figure 3). The trajectories that form products in the initial collision are show little selectivity, affording a 35:65 ratio of **Mark**:**anti-Mark** within 300 fs. Trajectories forming product after ∼500 fs involve one to five cycles of collision and recoil (defined as the C–B distance increasing to over 2.0 Å, typically to 2.5–3.0 Å on the first bounce), but only 5.4% of the 15.3% total **Mark** product arises after recoil. Most (72%) of the **anti-Mark** product is formed after recoil.

**Figure 3:**
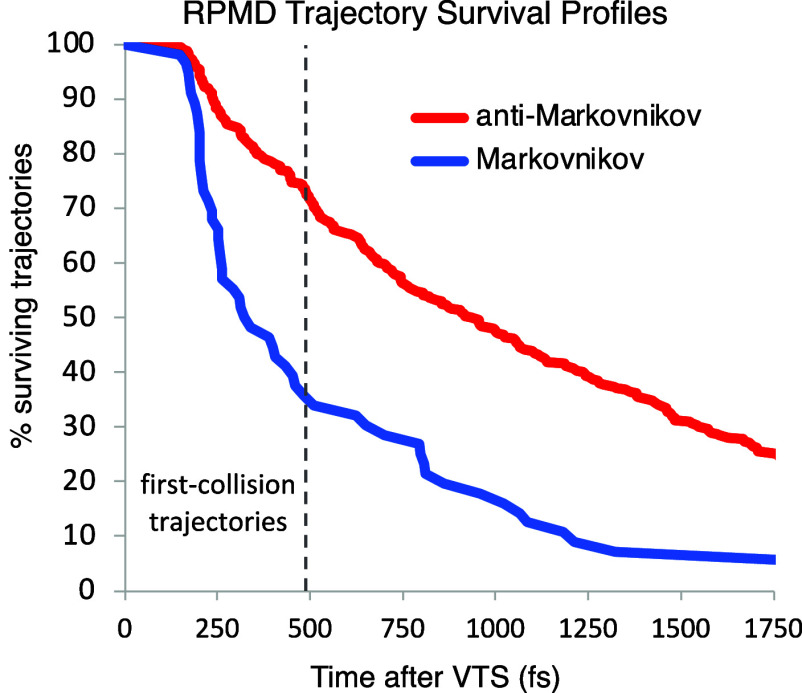
Survival profiles for RPMD trajectories for the reaction of BH_3_ with propene. Note that on an absolute scale, the anti-Markovnikov trajectories outnumber the Markovnikov by a factor of ∼6. See the SI for an expanded version and additional survivor profiles.

Figure 4 shows milestone structures along the path for a typical first-collision formation of the Markovnikov product (see the SI for additional examples). The depiction exhibits each of the RPMD beads for each atom and may be viewed as reflecting the delocalization of the nuclei.^7^ The mutual approach of the reactants accelerates after the **VTS** (0 fs) as the structure descends into the energy well of **INT**. The anti-Markovnikov/Markovnikov orientation of the BH_3_ hydrogens in this approach appears dominated by random chance; as in the example at 200 fs, 46% of the trajectories have an H atom first approach the terminal carbon to within 2.3 Å. After passing through the area of **INT** at ∼210 fs, a notable feature of the **Mark** RPMD trajectories is that the atomic beads for the transferring H become more diffuse. That is, the volume of a sphere defined by the average distance of the H beads from the centroid increases by on average ∼45% as the H is transferred. The **anti-Mark** trajectories exhibit little transferring H-bead expansion (∼16%), plausibly because the small (∼0.2) kcal/mol barrier for **anti-Mark** formation on the adiabatic ground-state surface involves less tunneling. The association of bead-volume with tunneling is supported by trajectories conducted at 10 K starting at **INT**. Classical trajectories cannot cross the barrier at 10 K, but RPMD trajectories readily do so as they exhibit bead-volume expansions of up to a factor of 5.

**Figure 4:**
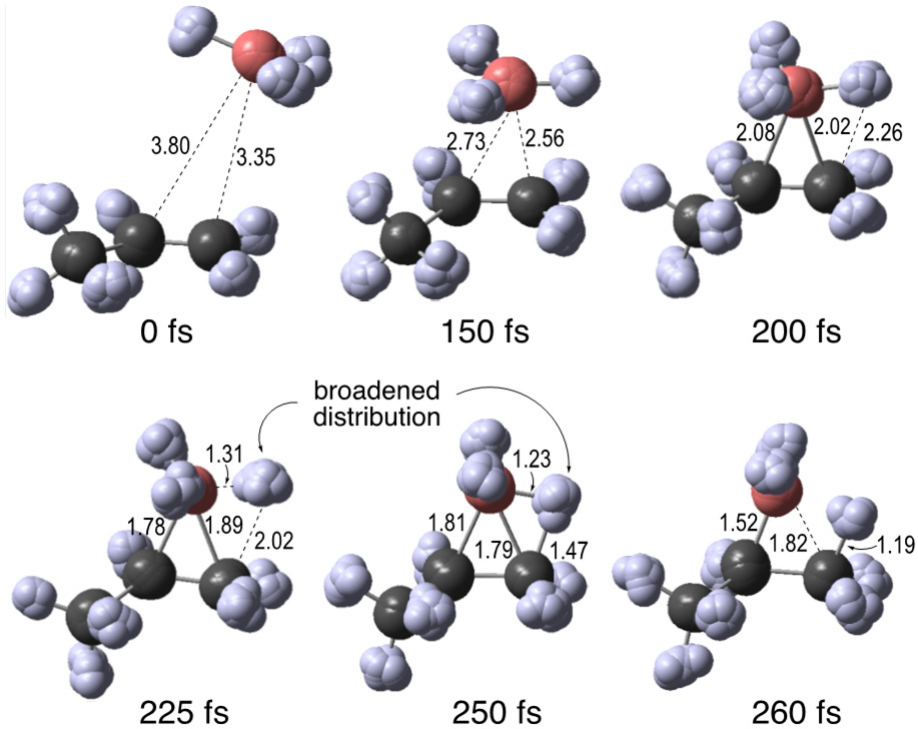
Milestone structures from an RPMD trajectory affording **Mark**, showing all beads and bonds between centroids.

Our interpretation of these observations is that tunneling in the collision of reactants plays a substantial role in the regioselectivity of hydroboration. The diminished **Mark** product with BD_3_ in both the experiment and the RPMD trajectories supports this idea. The decreased **Mark** in BD_3_ trajectories was the result of a drastic falloff (from 10% to 4.1%) in the fraction that affords **Mark** on collision. Both **Mark** and **anti-Mark** formation are slowed with BD_3_, but the **Mark** is slowed to a much greater extent (median trajectory times increase from 340 to 750 fs for **Mark**, 821 to 992 fs for **anti-Mark**).

Tunneling would be expected to favor the **Mark** product because of its greater adiabatic ground-state barrier (2.5 kcal/mol) compared to the 0.2 kcal/mol barrier for **anti-Mark** formation from **INT**. This general expectation is supported by the statistical CVT/SCT calculation, though this underestimates the magnitude of the effect.

At first glance, the most perplexing observation is that tunneling appears substantial in reactions where the energy available from formation of **INT**, ∼ 11 kcal/mol, is far greater than that required to overcome the barrier for **Mark** formation. Why should tunneling matter when there is enough energy to exceed the barrier anyway? It is a tautology, however, that when a larger proportion of random collisions are forming the **Mark** product, a larger diversity of trajectories are involved, meaning that trajectories straying further from the minimum-energy path have become productive. This idea is supported by an examination of the collisionally reactive trajectories; as the H atom transferring from B to C_1_ passes the saddle-point distance of 1.7 Å, a broader range of C_1_–B (centroid) distances is seen for the 16-bead BH_3_ trajectories (σ = 0.08 Å) than for the classical trajectories (σ = 0.04 Å). This suggests that tunneling is increasing the collisional cross-section by facilitating nonideal productive trajectories.

With larger alkenes in solution, experimental observations suggest the importance of both intermolecular energy transfer and intramolecular energy redistribution on hydroboration selectivity.^4b^ Nuclear quantum effects should be important in both phenomena, as well as the hydroboration process itself, and a full understanding of experiment will require models that include these effects. Like many models in chemistry, RPMD cannot be fully justified theoretically, and this is doubly true for nonequilibrium dynamics. Nonetheless, RPMD’s success here suggests its unique value for other reactions involving a combination of nonstatistical dynamics and nuclear quantum effects, and it should now be practical for the study of solution reactions. Such studies are planned.

## Supporting Information Available

Complete descriptions of experimental and computational procedures, complete trajectory results, discussions of energy conservation, additional milestone structures and survivor profiles (PDF)
Movie showing example RPMD trajectories (MP4)
Movie showing example RPMD trajectories (MP4)
Movie showing example RPMD trajectories (MP4)
