# Supplementary material for: Nuclear Quantum Effects on the Nature of Hydroboration Selectivity: Experimental Effects of First-Collision Tunneling
Source: J Am Chem Soc. 2024 Sep 16;146(38):25907–11. doi: 10.1021/jacs.4c09306 (PMC11440546; doi:10.1021/jacs.4c09306)
Supplement: Supplementary file 1 — ja4c09306_si_001.pdf [file ja4c09306_si_001.pdf]

SUPPORTING INFORMATION

Nuclear Quantum Effects on the Nature of Hydroboration Selectivity. Experimental Effects of First-Collision Tunneling

Christoph E. Bracher, Connor J. Allen, and Daniel A. Singleton*

Department of Chemistry, Texas A&M University, 3255 TAMU,
College Station, Texas 77843, United States
singleton@chem.tamu.edu

Table of Contents

Experimental Procedures S1
General Methods S1
Deuteroboration of 1-Hexene S2
Computational Procedures and Extended Results S3
General Procedures S3
DFT Method Choice S4
Complete Trajectory Results and Comments S4
Discussion of the Application of RPMD to a Non-equilibrium Reaction. S5
Additional Figures Showing Milestone Structures S7
Expanded Figure 3 .... S8
Markovnikov Survivor Profiles Places on the Same Scale S9
Programs for Calculations and Sample Input Files S10
Modified RPMDrate S10
Patch Files S13
gforce.py S19
input.py S22
A Sample Slurm Script S23
Helper and Data-Analysis Programs S25

Experimental Procedures

General Methods

Oven-dried glassware was flushed with N_2 while hot before use. Standard syringe-and-septum techniques were employed, with reactions carried out under a positive pressure of N_2. THF (Thermo Scientific, CAS 109-99-9, lot T06J808) was distilled from sodium-benzophenone ketyl just before use. Hexene was used as received from Sigma-Aldrich (CAS 592-41-6, lot SHBB9937V) and Oakwood (lot 098845P10G-1). Sodium borodeuteride was used as received from Oakwood Chemical (CAS 15681-89-7, lot 098904R05F, new bottles). Boron trifluoride diethyl etherate was used as received from Sigma-Aldrich (CAS 109-63-7, lot SHBF7337V). ^{1}H NMR spectra were recorded on a 500 MHz Varian NMRS 500 using C_6D_6 as solvent.

Deuteroboration of 1-Hexene

The procedure here is designed to closely mimic that reported for the hydroboration of alkenes in main-text reference 4b. The regiochemistry result in reference 4b was found to be reproducible.

Example Procedure. A flask containing 0.46 g (11 mmol) of $NaBD_4$ and 9 mL of THF was submerged in a room temperature water bath and stirred rapidly. To this mixture was added dropwise 1.25 mL (10.0 mmol) of $BF_3{\cdot}OEt_2$, and the solution was stirred for 5 min after the addition was complete. A mixture of 0.084 g (1.00 mmol) of 1-hexene in 1 mL of THF was then added dropwise over a 5-min period. After 5 min of additional stirring, ice was added to the water bath, and 5 mL of water was added, initially by a slow dropwise addition until gas evolution subsided (~0.5-1 mL), then the rest of the water was added more rapidly. To this mixture was added 4 mL of 3M aqueous NaOH followed by 4 mL of 30% H_2O_2. The ice bath was removed, and the resulting mixture was stirred at room temperature for 2 h. Solid NaCl was added to saturation, and the layers were separated. The aqueous phase was extracted with three 10-15 mL portions of THF. The combined organic phases were rinsed twice with 10 mL of brine, once with a mixture of 5 mL brine and 5 mL saturated aqueous Na_2SO_4, then once more with 10 mL of brine. The organic layer was dried over $MgSO_4$ and concentrated on a rotary evaporator. The wet residue was diluted with 2 mL of C_6D_6 and dried with Na_2SO_4, and the clear liquid was transferred with a pipette and concentrated again. The resulting residue was diluted with C_6D_6, filtered through glass wool, and analyzed directly by ^{1}H NMR spectroscopy. The product ratios were measured by comparison of the doublet of 1:1:1 triplets of the deuterated methyl group of the secondary alcohol (**Mark**) product and the doublet of triplets of the methylene protons on C2 of the primary alcohol (**anti-Mark**) product.

A sample spectrum of the deuterated hexanol product is shown in Figure S1. In some spectra, broad peaks were observed at δ 3.26 and 1.63 ppm, and their interference by partial overlap with the **anti-Mark** peak varied with the concentration of the solution. For spectral processing, the **anti-Mark** peak was phased by comparison of flanking integrals where the baseline was free of signal by iteratively using the zero-order autophasing command provided by VNMRJ and adjusting the LP parameter. In some cases, the RP parameter had to be adjusted slightly further to achieve a flat baseline in the area of the **Mark** peak.

The baseline level was then subjected to a zeroth-order correction such that integral regions flanking the **Mark** peak were as flat as possible. The idea here is that baseline effects have a larger proportionate influence on a small peak than on a large peak, so the baseline was adjusted to minimize the effect on the small **Mark** peak. In principle, the baseline for both peaks could be made flat using a higher-order baseline correction, but we have long rejected the use of higher-order corrections because they give too much leeway to the experimentalist to make choices that affect the result. Instead, the integration for the large **anti-Mark** peak was adjusted by a "Corrector" value based on the integration of the baseline in a downfield area with no signal. The percentage of the **Mark** product was calculated as 100 x (**Mark**) / (**anti-Mark** – Corrector + **Mark**). The 95% confidence ranges were calculated from the standard deviations and number of measurements in a normal way (See: http://www.iupac.org/publications/analytical_compendium/Cha02sec3.pdf).

Approximately 3% of the product mixture was unlabeled due to protio contamination in the BD_3. Allowance for this would slightly decrease the amount of **Mark** arising from BD_3 and slightly increase the magnitude of the KIE in the main text, but it was judged that too many

assumptions were required to justify a specific small numerical correction.

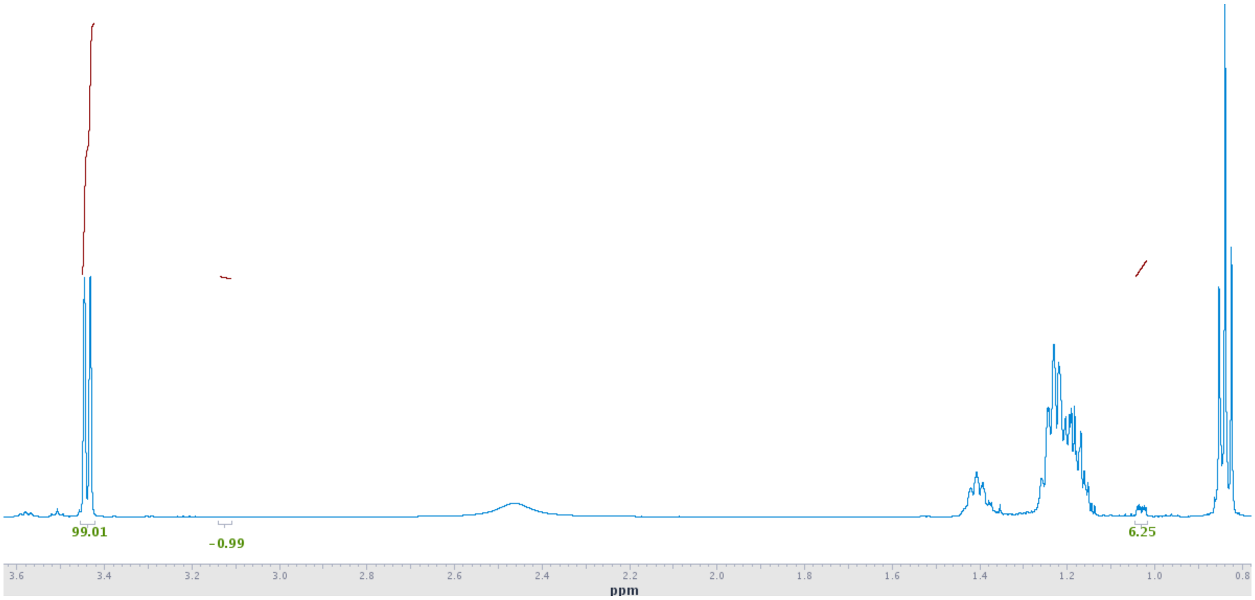

Figure S1. Sample ^{1}H NMR spectrum of the product mixture from deuteroboration of 1-hexene.

Table S1. NMR data for the determination of product ratios for the deuteroboration of 1-hexene.

Sample	**anti-Mark**	Corrector	**Mark**	% **Mark**
1	95.71	-4.29	5.64	5.3
2	99.014	-0.994	6.247	5.9
3	98.906	-1.097	6.085	5.7
4	97.92	-2.079	6.907	6.5
5	97.42	-2.582	6.651	6.2
6	98.62	-1.378	6.806	6.4
7	99.00	-1.000	6.776	6.3
Average				6.1
Std dev				0.41
95% confidence interval				0.38

Computational Procedures and Extended Results

General Procedures

A later section will describe in detail the modified RPMDrate code employed along with input options. Almost all of the regular DFT structures and energies described in the current study have been previously reported in main text reference 4. The exception to this is that further methods validation studies were carried out, as described below. These studies employed Gaussian16,[1] with default procedures (though the geometries had been optimized employing "tight" convergence criteria).

Calculations of rate constants and KIEs including small-curvature tunneling (SCT) employed the GAUSSRATE / POLYRATE set of programs.[2,3] Main text reference 4b included sample POLYRATE input files, and the only new POLYRATE calculations here are those

employing isotopically labeled borane in a standard way. CVT/SCT predictions that are compared with trajectory results are purely B3LYP/6-31G*, while CVT/SCT predictions that are compared with experiment are based on interpolated single-point energies (VTST-ISPE), using CCSD(T)/aug-cc-pvtz single-point energies by the method described in 4b.

The RRKM-ME calculations including an approximate SCT correction were carried out by the methods described in main text reference 4b.

A series of short helper and data-analysis programs was used to aid the running of trajectories and to analyze the data. These are described and listed in a later section.

DFT Method Choice

The 2009 paper of Oyola and Singleton (reference 4a in the main text) had examined a total of 61 method / basis set combinations by comparing single-point energies for structures with potential energies obtained from CCSD(T)/aug-cc-pvtz calculations. This was done for a series of structures in the area of the **TSA** / **TSM**, with the priority of having the most accurate surface in this area. Of all methods investigated, B3LYP/6-31G* energies most closely matched the CCSD(T)/aug-cc-pvtz energies in this area, with an RMS deviation of 0.19 kcal/mol. For the current paper, the original exploration was supplemented using M06-2X, M11, MN15, and ωB97xD functionals, with differing basis sets. Each of these methods exhibited RMS errors > 1.0 kcal/mol in the critical region. B3LYP/6-31G* does somewhat underestimate the exothermicity of formation of the **INT** from separate propene / BH_3 (downhill in potential energy by 11.6 kcal/mol B3LYP, 13.5 kcal/mol CCSD(T)) while the M06-2X, M11, MN15, and ωB97xD functionals all overestimate this energy by ~3-4 kcal/mol. B3LYP/6-31G* would not be our aesthetic choice for a calculation in 2024 but, in this system, its (arguably accidental) accuracy in the critical area of the reaction surface led us to maintain its use for the current paper. Including GD3 empirical dispersion with B3LYP/6-31G* improved somewhat the energy of propene-BH_3 interaction (to 14.1 kcal/mol) but also slightly worsened the fit with the CCSD(T) surface (to an RMS error of 0.28 kcal/mol.

Complete Trajectory Results and Comments

As described in the main text, the RPMD trajectories were first equilibrated and thermostated for 298.15 K in the area of **VTS** or **INT**. This was done by applying loose harmonic constraints (delineated in the later code listing) during the equilibration, then releasing the constraints for snapshots extracted every 50 fs. Autocorrelation among the sequentially extracted trajectories was found to be negligible. The initialization of trajectories for snapshots employed the same random Maxwell sampling process[4] and code that has been used previously with RPMDrate in its many kinetics calculations.

For practicality, a relatively long 0.5 fs time step was employed. For the temperature employed, this leads to somewhat jerky bead motion but no discernable anomalies. Exploratory trajectories using a 0.2 fs time step gave results that were not statistically different from those using 0.5 fs steps.

It is notable that ~40-60% of the trajectories released from **VTS** returned to reactants, as judged by stopping criteria of C_3—B > 3.5 Å and C_2—B > 3.9 Å. This suggests that the geometry of **VTS**, originally obtained from a "downhill" statistical process (see main text reference 4b), is a reasonably good approximation of a 'true' variational transition state.

The RPMD-predicted product mixture with 15.3% **Mark** obviously does not match the experimental product mixture with 10.7% ± 0.5% **Mark**. This difference perhaps needs no

explanation, considering the gas-phase nature of the calculation (and that the trajectories are B3LYP/6-31G* with no high-level energies (as possible for the statistical calculations)). However, we would speculate that solution collision is dampened by the solvent, decreasing the available excess energy. We believe that RPMD trajectories in a ball of low-level solvent molecules would now be reasonably practical to answer this kind of question.

Table S2. Trajectory results.

Reaction	Beads	**anti-Mark** (median time)	**Mark** (median time)	>3000 fs	Returned reactants	% **Mark**[a]
Propene/BH_3 from **VTS**	1	251 (946 fs)	32 (423 fs)	44	225	9.8%
	4	83 (1083 fs)	12 (315 fs)	14	158	11.0%
	16	287 (821 fs)	56 (339 fs)	23	386	15.3%
	32	1 (497 fs)	1 (272 fs)	-	1	
Propene/BH_3 from **INT**	16	142 (504 fs)	3 (305 fs)	-	-	2.1% (2.5% CVT/SCT)
Propene/BD_3 from **VTS**	1	281 (946 fs)	34 (746 fs)	69	318	8.9%
	16	261 (992 fs)	32 (553 fs)	43	355	9.5%

[a]Calculated as **Mark**/(**Mark** + **anti-Mark** + >3000 fs). Because only ~2% of the trajectories taking greater than 2000 fs but less than 3000 fs afforded **Mark**, all of the trajectories reaching a 3000-fs time limit were assigned as **anti-Mark**. Assigning 2% of these trajectories to **Mark** makes no significant difference in the overall results.

Discussion of the Application of RPMD to a Non-equilibrium Reaction.

There are two interrelated potentially serious problems in the application of RPMD to the type of non-equilibrium reaction involved in hydroboration. The first, as described in the main text, is the issue of how to approximate the temperature of the internal ring-polymer modes. As the reactants fall from **VTS** to **INT**, there is excess energy generated, and it is non-statistically distributed. As we described, the approximation employed was to simply use the thermostated temperature in an ordinary way without modification. There is certainly no strict justification for this (a word used to describe the attitude of the quantum dynamics community is “desperation” (see main text reference 9a), but one may consider the errors engendered. At sufficiently short times, this approximation is no different than that used in the recrossing trajectory calculation in an RPMD rate calculation (main text references 8 and 9). At longer times, however, the most serious possible error would be that the excess energy could be redistributed into the imaginary modes due to coupling between the polymer springs and the dynamics of the centroid (as happens for spurious resonances when RPMD is used to predict IR spectra). This would in effect heat the imaginary dimensions and cool the real, and it is this redistribution that is the second potentially serious problem with using RPMD for non-equilibrium reactions. We therefore endeavored to

examine the time scale for this energy redistribution.

With ordinary classical trajectories, the examination of energy conservation is straightforward. Indeed, in a one-bead simulation starting from **VTS**, the total of the potential energy and the nuclear kinetic energy was constant within 0.05 kcal/mol over the course of 2390 fs.

With multiple beads, however, a difficulty arises as to how to divide the energy between the real and imaginary. The total is constant, but if one tries to examine the amount of energy in the real dimensions by summing either the centroid potential energy and + centroid kinetic energy or the average bead potential energy + centroid kinetic energy, the total varies. Figure S2 shows an example plot of the average bead potential energy + centroid kinetic energy for a trajectory that formed **anti-Mark** and that was extended for an additional 1000 fs after **anti-Mark** formation. The total energy varies in multiple ways: a. over short times with a standard deviation of ~2 kcal/mol; b. by ~2 kcal/mol as the π-complex is formed; and c. after the reaction occurs, so that the final energy is about 4 to 5 kcal/mol below the initial total energy. A way to understand these changes is that the 'total' energy does not fully include the zero-point energy, and the zero-point energy changes with structure as the trajectory progresses. In fact, the harmonic zero-point energy for **anti-Mark** differs from that for **VTS** by 3.9 kcal/mol.

The formation of **anti-Mark** is downhill in potential energy from **VTS** by 29.9 kcal/mol. If this excess energy was being lost into the very high number of imaginary dimensions at a rapid rate, the total real energy would be expected to drop by >10 kcal/mol from the gain in potential energy in the springs. This is not happening; the energy redistribution after **anti-Mark** formation is, we estimate, less than 2 kcal/mol over 1000 fs. On the time scale of the median trajectory forming **Mark** or **anti-Mark** from **VTS** through **INT** (downhill in potential energy from **VTS** by 9.7 kcal/mol), the energy redistribution appears negligible. These observations ameliorate the most obvious concern about the application of RPMD to the current reaction.

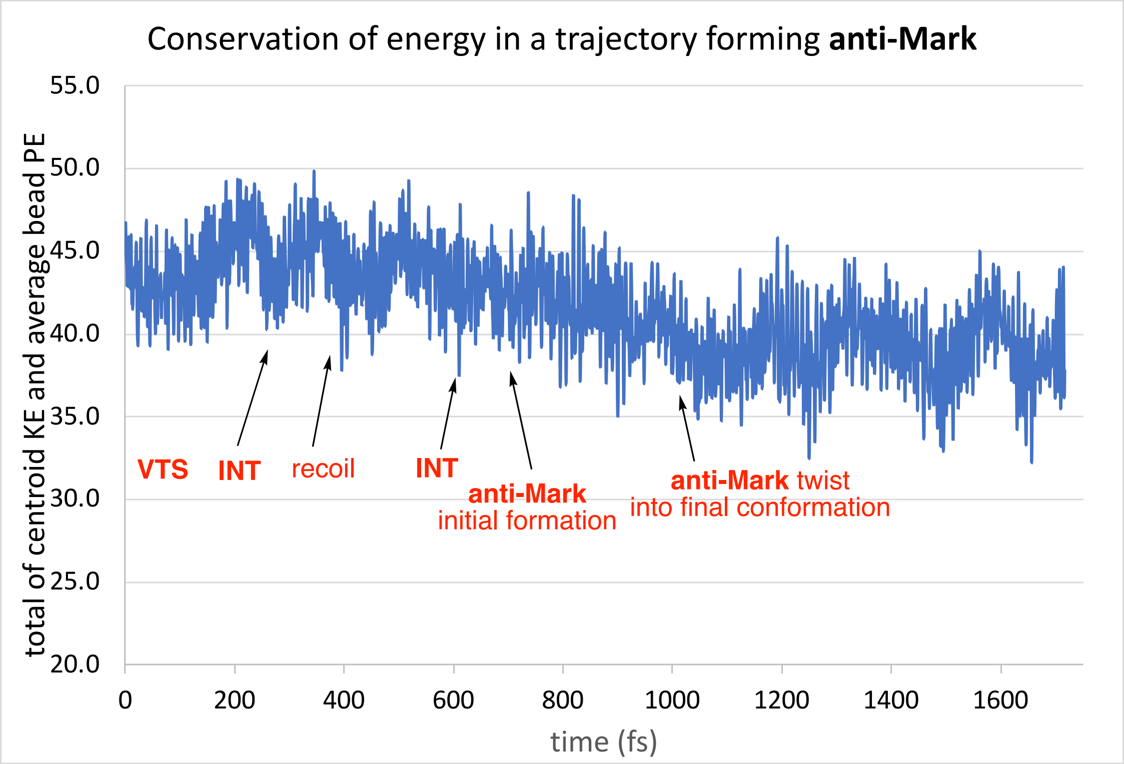

Figure S2. Sample graph of the total of the centroid kinetic energy and the average bead potential energy for an extended trajectory run forming the **anti-Mark** product.

Additional Figures Showing Milestone Structures

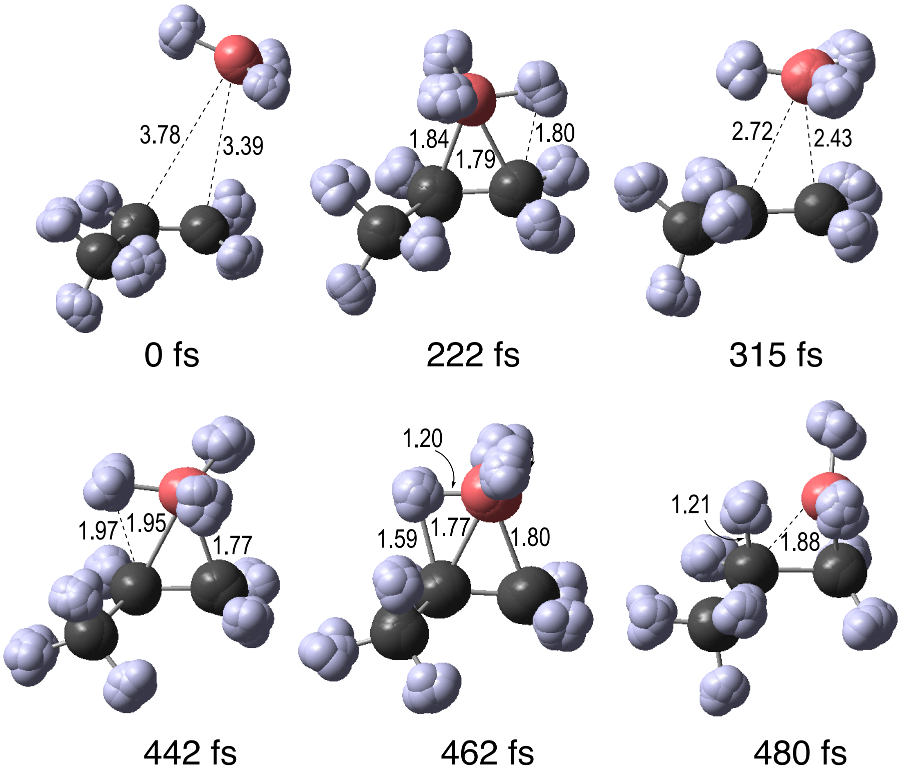

Figure S3. Milestone structures from a trajectory forming **anti-Mark** after the reactants initially approach in a manner oriented to form **Mark** (222 fs), then recoil (315 fs) and come back together (442 fs) in an orientation suitable for **anti-Mark** formation.

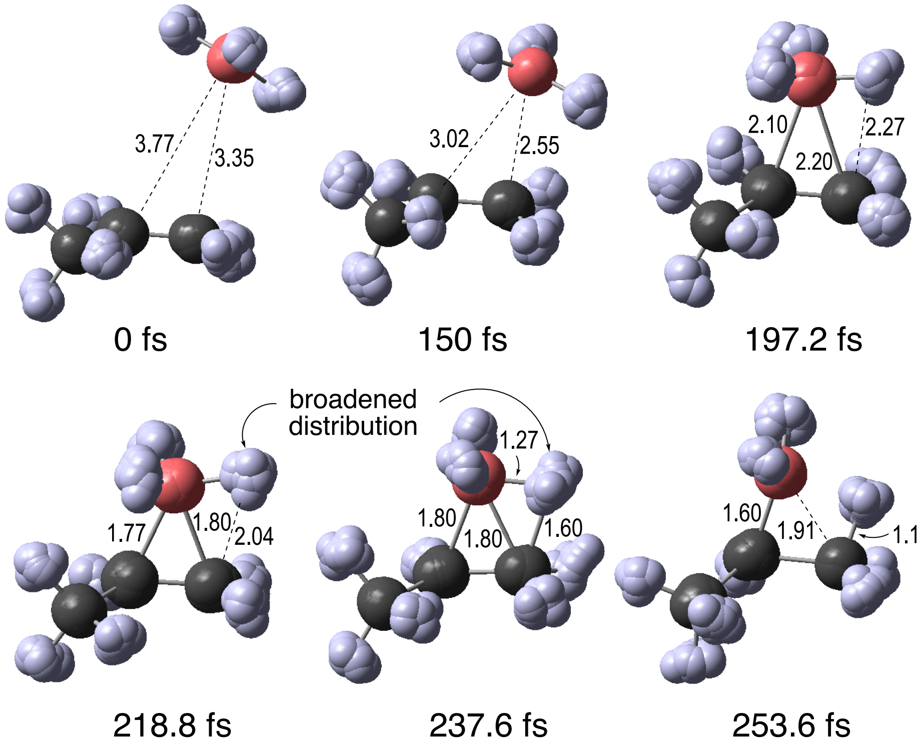

Figure S4. Milestone structures from a trajectory forming **Mark** in the initial collision.

Expanded Figure 3

Figure 3 in the main text was limited in its domain to improve its readability. Figure S5 shows a version with a complete domain up to the time limit of 3000 fs. As discussed above, trajectories that had not completed by 3000 fs were assigned to **anti-Mark**. It is expected that a small portion of these would afford **Mark**, but a correction for this would make little difference in the analysis.

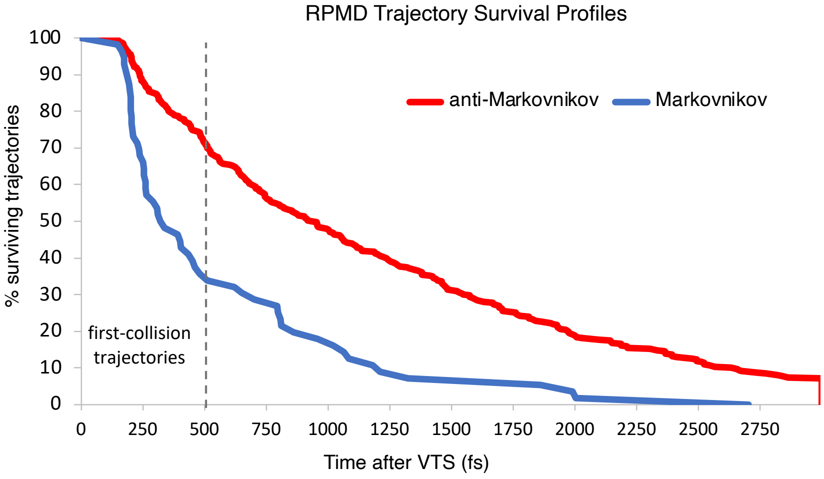

Figure S5. Domain-expanded version of main-text Figure 3.

Markovnikov Survivor Profiles Places on the Same Scale

To show the difference between Markovnikov product formation 16-bead BH_3, 16-bead BD_3, and classical survivor profiles, Figure S6 exhibits these profiles on the same scale, that is, as a percentage of the total trajectories. This figure illustrates how the first-collision **Mark** formation dominates the 16-bead BH_3 trajectories, while they are decreased substantially for BD_3, and are a very factor for the classical trajectories.

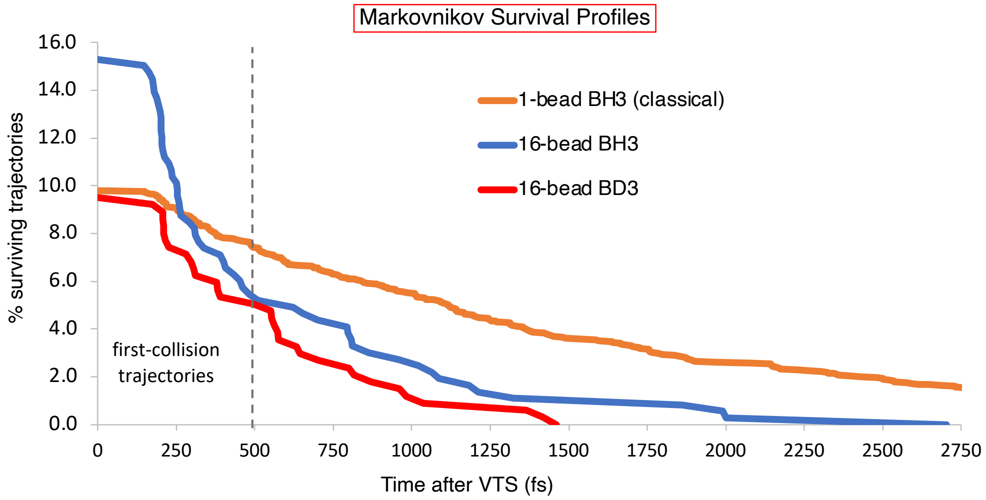

Figure S6. Survivor profiles for Markovnikov product formation for 16-bead BH_3, 16-bead BD_3 and classical trajectories placed on the same scale as the percentage of the total number of reactive trajectories.

Programs for Calculations and Sample Input Files

To facilitate the straightforward reproduction of the research, the section below describes in detail how to obtain and set up the modified RPMDrate code along with specific sample input and job submission files. We also include the code for a number of complex helper scripts that were developed to extract computational results and to set up computations for additional analyses or graphical depiction.

The authors request that users of these programs inform them at singleton@chem.tamu.edu for notifications of bug fixes and updated features and to allow tracking of the broader impacts of the programs.

Modified RPMDrate

The program RPMDrate was modified substantially for the purpose at hand. We describe here a step-by-step process by which one can obtain and execute the modified RPMDrate that was employed here.

1. The original code can be obtained from github.com at https://github.com/GreenGroup/RPMDrate
   You will start with the directory RPMDrate-master which contains as a listing:
   documentation examples Makefile README.rst rpmdrate rpmdrate.py setup.py tests
2. The most difficult part of installing RPMDrate is establishing a compatible combination of python modules, compilers, and libraries. The original RPMDrate installation instructions (https://greengroup.mit.edu/rpmdrate-installation) are largely correct, but can no longer be considered complete. Modern versions of python (3.6+) will not run the rpmdrate code without modification, so using python 2 in the context of a conda environment (e.g. conda/anaconda/miniconda) is indicated. RPMDrate uses various python modules and functions, including some that have since been deprecated and removed, requiring module versions within a specific range. No attempt was made to determine all such dependencies, but the libraries and modules we used are given here.

FFTW = "3.3.10"	PMIx = "4.2.4"	libfabric = "1.18.0"
FFTW.MPI = "3.3.10"	UCC = "1.2.0"	libpciaccess = "0.17"
GCC = "12.3.0"	UCX = "1.14.1"	libxml2 = "2.11.4"
GCCcore = "12.3.0"	XZ = "5.4.2"	numactl = "2.0.16"
Miniconda3 = "23.5.2-0"	binutils = "2.40"	zlib = "1.2.13"
OpenMPI = "4.1.5"	hwloc = "2.9.1"	
OpenSSL = "1.1"	libevent = "2.1.12"	

The 2023.1.0 version of the intel-compilers was used.

To create the conda environment with the correct packages, it was necessary to first add the conda-forge repository:

conda config --add channels conda-forge

The conda environment contains the following packages:

# Name	Version	Build	Channel
_libgcc_mutex	0.1	conda_forge	conda-forge
_openmp_mutex	4.5	2_gnu	conda-forge
ca-certificates	2024.2.2	hbcca054_0	conda-forge
certifi	2019.11.28	py27h8c360ce_1	conda-forge

```
ld_impl_linux-64          2.40                 h41732ed_0    conda-forge
libblas                   3.9.0          21_linux64_openblas    conda-forge
libcblas                  3.9.0          21_linux64_openblas    conda-forge
libffi                    3.2.1           he1b5a44_1007    conda-forge
libgcc-ng                 13.2.0               h807b86a_5    conda-forge
libgfortran-ng            13.2.0               h69a702a_5    conda-forge
libgfortran5              13.2.0               ha4646dd_5    conda-forge
libgomp                   13.2.0               h807b86a_5    conda-forge
liblapack                 3.9.0          21_linux64_openblas    conda-forge
libopenblas               0.3.26         pthreads_h413a1c8_0    conda-forge
libsqlite                 3.45.1               h2797004_0    conda-forge
libstdcxx-ng              13.2.0               h7e041cc_5    conda-forge
libzlib                   1.2.13               hd590300_5    conda-forge
ncurses                   6.4                  h59595ed_2    conda-forge
numpy                     1.16.5           py27h95a1406_0    conda-forge
openssl                   1.1.1w               hd590300_0    conda-forge
pip                       20.1.1             pyh9f0ad1d_0    conda-forge
python                    2.7.15        h5a48372_1011_cpython    conda-forge
python_abi                2.7                    1_cp27mu    conda-forge
quantities                0.12.0                  py27_0    conda-forge
readline                  8.2                  h8228510_1    conda-forge
setuptools                44.0.0                  py27_0    conda-forge
sqlite                    3.45.1               h2c6b66d_0    conda-forge
tk                        8.6.13        noxft_h4845f30_101    conda-forge
wheel                     0.37.1             pyhd8ed1ab_0    conda-forge
zlib                      1.2.13               hd590300_5    conda-forge
```

In yml format:

```
name: 27rpmd
channels:
  - conda-forge
  - defaults
dependencies:
  - _libgcc_mutex=0.1=conda_forge
  - _openmp_mutex=4.5=2_gnu
  - ca-certificates=2024.2.2=hbcca054_0
  - certifi=2019.11.28=py27h8c360ce_1
  - ld_impl_linux-64=2.40=h41732ed_0
  - libblas=3.9.0=21_linux64_openblas
  - libcblas=3.9.0=21_linux64_openblas
  - libffi=3.2.1=he1b5a44_1007
  - libgcc-ng=13.2.0=h807b86a_5
  - libgfortran-ng=13.2.0=h69a702a_5
  - libgfortran5=13.2.0=ha4646dd_5
  - libgomp=13.2.0=h807b86a_5
  - liblapack=3.9.0=21_linux64_openblas
  - libopenblas=0.3.26=pthreads_h413a1c8_0
```

- libsqlite=3.45.1=h2797004_0
- libstdcxx-ng=13.2.0=h7e041cc_5
- libzlib=1.2.13=hd590300_5
- ncurses=6.4=h59595ed_2
- numpy=1.16.5=py27h95a1406_0
- openssl=1.1.1w=hd590300_0
- pip=20.1.1=pyh9f0ad1d_0
- python=2.7.15=h5a48372_1011_cpython
- python_abi=2.7=1_cp27mu
- quantities=0.12.0=py27_0
- readline=8.2=h8228510_1
- setuptools=44.0.0=py27_0
- sqlite=3.45.1=h2c6b66d_0
- tk=8.6.13=noxft_h4845f30_101
- wheel=0.37.1=pyhd8ed1ab_0
- zlib=1.2.13=hd590300_5

3. Once the compiler and conda environment is correct, the program can be compiled with:
   python setup.py build_ext --inplace
4. Before applying any patches, it is best to make sure that one or more of the examples provided with the RPMDrate code can be run successfully. As an example, go into examples/H+CH4, then compile the potential energy surface with:
   make
   then try out the program with this command from the RPMDrate-master directory:
   python rpmdrate.py examples/H+CH4/input.py 300.0 4 -p 4
   If this runs for a few minutes without error then one can proceed with the patches.
5. Install the patch files from the next section, with the names given, in the RPMDrate-master directory. Then run these patch commands:
   patch -u rpmdrate/_main.pyf -i _main.pyf.patch
   patch -u rpmdrate/_main.f90 -i _main.f90.patch
   patch -u rpmdrate/input.py -i input.py.patch
   patch -u rpmdrate.py -i rpmdrate.py.patch
   patch rpmdrate/main.py -i main.py.patch
   patch -u rpmdrate/surface.py -i surface.py.patch
   and then recompile with:
   make clean
   make
   It should be noted that these modifications make RPMDrate bypass its normal umbrella sampling process (not of interest here), making it unsuitable for its original use.
6. Create a directory “BH3” in the RPMDrate-master directory, then put into the files gforce.py and input.py from a later section. (There are two distinct files named input.py, one that is in the rpmdrate directory and the other that is in the BH3 directory. The two should not be confused.) The gforce.py file should be modified on lines 33 and possibly line 40 to allow for the local number of processors available and scratch directory.

7. The Gaussian 16 calculations are set up by gforce.py to make use of a checkpoint file from a previous Gaussian calculation on the starting geometry in input.py. For a single-bead calculation job, this checkpoint file should be named bead0.chk. With additional beads, this file should be copied to bead1.chk, bead2.chk, … up to the number of beads. These checkpoint files should be in the working directory used for the Gaussian calculations, but may be in the RPMDrate-master directory for an initial trial.
8. With everything in place and with Gaussian 16 initiated in a system-dependent way, run the command:
   python -u rpmdrate.py BH3/input.py 273.15 1
   from the RPMDrate-master directory to try out the program.
9. The adaptation of the modified RPMDrate to other problems requires straightforward modifications at lines 1148 and 1153 of _main.f90 to allow for the atoms in the system of interest, along with modifications of gforce.py and input.py (the version in the BH3 directory) to allow for the system under study, the constraints in the equilibration phase of the calculation, and data extraction from the output files.

Patch Files

Each of the files below should be named as shown and put into the RPMDrate-master directory, before running the patch commands shown in the previous section.

file _main.pyf.patch

```
--- RPMDrate-master/rpmdrate/_main.pyf          2024-05-19
17:49:08.873122000 -0500
+++ ../rpmdBD316aa/rpmdrate/_main.pyf          2024-05-17
17:53:40.846347000 -0500
@@ -3,12 +3,14 @@

 python module _main__user__routines
     interface
-        subroutine potential(q,v,dvdq,natoms,nbeads,info)
+        subroutine
potential(q,v,dvdq,natoms,nbeads,DASflag,t,info)
            double precision
dimension(3,natoms,nbeads),intent(in) :: q
            double precision
dimension(nbeads),intent(out),depend(nbeads) :: v
            double precision
dimension(3,natoms,nbeads),intent(out),depend(natoms,nbead
s) :: dvdq
            integer,
optional,intent(in),check(shape(q,1)==natoms),depend(q) ::
natoms=shape(q,1)
            integer,
optional,intent(in),check(shape(q,2)==nbeads),depend(q) ::
nbeads=shape(q,2)
+           integer, optional,intent(in) :: DASflag
+           double precision, optional,intent(in) :: t
            integer, intent(out) :: info
        end subroutine potential
    end interface
```

file _main.f90.patch

```
--- RPMDrate-master/rpmdrate/_main.f90          2024-05-19
17:49:08.850677000 -0500
+++ ../rpmdBD316aa/rpmdrate/_main.f90          2024-05-17
17:53:40.842991000 -0500
@@ -34,7 +34,7 @@
    double precision :: dt
    double precision :: beta
    double precision :: mass(MAX_ATOMS)
-   integer :: mode
+   integer :: mode, DASflag
    double precision :: pi = dacos(-1.0d0)

    ! The type of thermostat (1 = Andersen, 2 = GLE)
@@ -87,10 +87,16 @@
        double precision :: V(Nbeads), dVdq(3,Natoms,Nbeads)
        double precision :: xi, dxi(3,Natoms),
d2xi(3,Natoms,3,Natoms)
        double precision :: centroid(3,Natoms)
-       integer :: step, andersen_sampling_steps
+       integer :: step, andersen_sampling_steps, DASflag

        result = 0

+       DASflag = 7
+       ! open(unit=889,file='DASflag')
+       ! write(889,fmt='(I6)') DASflag
+       ! close(unit=889)
+       print*, "DAS in _main.f90 in Subroutine equilibrate"
+
```

```
        ! Set up Andersen thermostat (if turned on)
        andersen_sampling_steps = int(andersen_sampling_time
/ dt)
        if (thermostat .eq. 1) then
@@ -112,7 +118,7 @@

        call get_centroid(q, Natoms, Nbeads, centroid)
        call get_reaction_coordinate(centroid, Natoms,
xi_current, xi, dxi, d2xi)
-       call potential(q, V, dVdq, Natoms, Nbeads, result)
+       call potential(q, V, dVdq, Natoms, Nbeads, DASflag, t,
result)
        if (result > 0) then
           ! The initial position is unphysical, so abort
           result = -1
@@ -205,10 +211,15 @@
        double precision :: V(Nbeads), dVdq(3,Natoms,Nbeads)
        double precision :: xi, dxi(3,Natoms),
d2xi(3,Natoms,3,Natoms)
        double precision :: centroid(3,Natoms), vs, fs
-       integer :: step
+       integer :: step, DASflag

        result = 0
+       DASflag = 40
+       ! open(unit=889,file='DASflag')
+       ! write(889,fmt='(I6)') DASflag
+       ! close(unit=889)

+       print*, "DAS in _main.f90 in subroutine
recrossing_trajectory"
        if (save_trajectory .eq. 1) then
           open(unit=777,file='child.xyz')
           open(unit=888,file='child_centroid.xyz')
@@ -216,7 +227,7 @@

        call get_centroid(q, Natoms, Nbeads, centroid)
        call get_reaction_coordinate(centroid, Natoms,
xi_current, xi, dxi, d2xi)
-       call potential(q, V, dVdq, Natoms, Nbeads, result)
+       call potential(q, V, dVdq, Natoms, Nbeads, DASflag, t,
result)
        if (result > 0) then
           ! The initial position is unphysical, so abort
           result = -1
@@ -230,9 +241,17 @@
        do step = 1, steps
           call verlet_step(t, p, q, V, dVdq, xi, dxi, d2xi, Natoms,
Nbeads, &
              xi_current, potential, 0.d0, 0, result)
-          if (result .ne. 0) exit
+          if (result > 0) exit
+
           if (save_trajectory .eq. 1) call update_vmd_output(q,
Natoms, Nbeads, 777, 888)
           if (xi .gt. 0) kappa_num(step) = kappa_num(step) + vs
/ fs
+
+          ! DAS mod to try a signal from gforce
+          if (result < 0) then
+             print*,"result",result,"step",step
+             result = 0
+             exit
+          end if
        end do

        if (save_trajectory .eq. 1) then
@@ -261,14 +280,19 @@
        double precision :: V(Nbeads), dVdq(3,Natoms,Nbeads)
        double precision :: xi, dxi(3,Natoms),
d2xi(3,Natoms,3,Natoms)
        double precision :: centroid(3,Natoms)
-       integer :: step, andersen_sampling_steps
+       integer :: step, andersen_sampling_steps, DASflag

        result = 0
        actual_steps = 0
+       DASflag = 5
+       ! open(unit=889,file='DASflag')
+       ! write(889,fmt='(I6)') DASflag
+       ! close(unit=889)

        av = 0.0d0
        av2 = 0.0d0

+       print*, "DAS in _main.f90 in subroutine
umbrella_trajectory"
        ! Set up Andersen thermostat (if turned on)
        andersen_sampling_steps = int(andersen_sampling_time
/ dt)
        if (thermostat .eq. 1) then
@@ -290,7 +314,7 @@

        call get_centroid(q, Natoms, Nbeads, centroid)
        call get_reaction_coordinate(centroid, Natoms,
xi_current, xi, dxi, d2xi)
-       call potential(q, V, dVdq, Natoms, Nbeads, result)
+       call potential(q, V, dVdq, Natoms, Nbeads, DASflag, t,
result)
        if (result > 0) then
           ! The initial position is unphysical, so abort
           result = -1
@@ -401,10 +425,18 @@
        integer, intent(out) :: result

        double precision :: centroid(3,Natoms)
-       integer :: i, j
+       integer :: i, j, DASflag

        result = 0
+       DASflag = 6
+       if (constrain .eq. 1) then
+          DASflag = 11
+       end if
+       ! open(unit=889,file='DASflag')
+       ! write(889,fmt='(I6)') DASflag
+       ! close(unit=889)

+       ! print*, "DAS in _main.f90 in subroutine verlet_step"
        ! Update momentum (half time step)
        p = p - 0.5d0 * dt * dVdq

@@ -427,15 +459,15 @@
```

```
        ! If constrain is on, the evolution will be constrained to
the
        ! transition state dividing surface
-       if (constrain .eq. 1) call constrain_to_dividing_surface(p,
q, dxi, Natoms, Nbeads, xi_current, result)
-       if (result .ne. 0) return
+       ! DAS if (constrain .eq. 1) call
constrain_to_dividing_surface(p, q, dxi, Natoms, Nbeads,
xi_current, result)
+       ! DAS if (result .ne. 0) return

        ! Update reaction coordinate value, gradient, and
Hessian
        call get_centroid(q, Natoms, Nbeads, centroid)
        call get_reaction_coordinate(centroid, Natoms,
xi_current, xi, dxi, d2xi)

        ! Update potential and forces using new position
-       call potential(q, V, dVdq, Natoms, Nbeads, result)
+       call potential(q, V, dVdq, Natoms, Nbeads, DASflag, t,
result)
        if (result > 0) return
        if (mode .eq. 1) then
           call add_umbrella_potential(xi, dxi, V, dVdq, Natoms,
Nbeads, xi_current, kforce)
@@ -446,11 +478,18 @@
        p = p - 0.5d0 * dt * dVdq

        ! Constrain momentum again
-       if (constrain .eq. 1) call
constrain_momentum_to_dividing_surface(p, dxi, Natoms,
Nbeads)
+       ! DAS if (constrain .eq. 1) call
constrain_momentum_to_dividing_surface(p, dxi, Natoms,
Nbeads)

        ! Update time
        t = t + dt

+       ! DAS attempt to end loop early by signalling from
potential without
+       ! killing things
+       ! if (result < 0) then
+       !     result = 0
+       !     return
+       ! end if
+
     end subroutine verlet_step

     ! Update the positions and momenta of each atom in each
free ring polymer
@@ -476,6 +515,7 @@
        double precision :: beta_n, twown, pi_n, wk, wt, wm,
cos_wt, sin_wt, p_new
        integer :: i, j, k

+       ! print*, "DAS in _main.f90 in Subroutine
free_ring_polymer_step"
        ! Transform to normal mode space
        do i = 1, 3
           do j = 1, Natoms
@@ -560,6 +600,7 @@
        double precision :: mult, sigma, dsigma, dx, coeff
        integer, intent(out) :: info

+       print*, "DAS in _main.f90 in subroutine
constrain_to_dividing_surface"
        call get_centroid(q, Natoms, Nbeads, centroid)

        ! The Lagrange multiplier for the constraint
@@ -633,6 +674,7 @@
        double precision :: coeff1, coeff2, lambda
        integer :: i, j, k

+       print*, "DAS in _main.f90 in Subroutine
constrain_momentum."
        coeff1 = 0.0d0
        do i = 1, 3
           do j = 1, Natoms
@@ -697,6 +739,7 @@
        double precision :: delta
        integer :: i, j, k

+       print*, "DAS in _main.f90 in subroutine
add_umbrella_potential"
        delta = xi - xi_current

        ! Add umbrella potential
@@ -737,6 +780,7 @@
        double precision :: fs, fs2, log_fs, coeff1, coeff2, dhams
        integer :: i, j, k, i2, j2

+       print*, "DAS in _main.f90 in subroutine
add_bias_potential"
        fs2 = 0.0d0
        do i = 1, 3
           do j = 1, Natoms
@@ -833,11 +877,13 @@
                 end do
              end do
           end do
+          ! print*, "DAS in mode 1 of subroutine
get_reaction_coordinate xi =",xi
        elseif (mode .eq. 2) then
           ! Recrossing factor
           xi = xi_current * s1 + (1 - xi_current) * s0
           dxi = xi_current * ds1 + (1 - xi_current) * ds0
           d2xi = xi_current * d2s1 + (1 - xi_current) * d2s0
+          ! print*, "DAS in mode 2 of subroutine
get_reaction_coordinate xi =",xi
        else
           write (*,fmt='(A,I3,A)') 'Invalid mode ', mode, '
encountered in get_reaction_coordinate().'
           stop
@@ -1098,23 +1144,34 @@
        double precision, intent(in) :: q(3,Natoms,Nbeads)
        integer, intent(in) :: beads_file_number,
centroid_file_number
        integer :: j, k
+       ! DAS
+       character(len=13) :: atomlist

        double precision :: centroid(3,Natoms)
```

```
+       ! DAS
+       atomlist = "CCCBHHHHHHHHH"
+
        call get_centroid(q, Natoms, Nbeads, centroid)

        write(beads_file_number,fmt='(I6)') Natoms * Nbeads
        write(beads_file_number,fmt='(A)')
        do j = 1, Natoms
           do k = 1, Nbeads
-              write(beads_file_number,fmt='(I4,3F11.6)') j,
q(1,j,k), q(2,j,k), q(3,j,k)
+              ! DAS modified to get Angstroms out instead of
bohr and labeled
+              ! with element symbols
+              write(beads_file_number,fmt='(A,3F11.6)')
atomlist(j:j), 0.529177*q(1,j,k), &
+                  0.529177*q(2,j,k), 0.529177*q(3,j,k)
           end do
        end do

        write(centroid_file_number,fmt='(I6)') Natoms
        write(centroid_file_number,fmt='(A)')
        do j = 1, Natoms
-           write(centroid_file_number,fmt='(I4,3F11.6)') j,
centroid(1,j), centroid(2,j), centroid(3,j)
+           ! DAS modified to get Angstroms out instead of bohr
and labeled with
+           ! element symbols
+           write(centroid_file_number,fmt='(A,3F11.6)')
atomlist(j:j), 0.529177*centroid(1,j), &
+               0.529177*centroid(2,j), 0.529177*centroid(3,j)
        end do

    end subroutine
@@ -1180,9 +1237,9 @@

        if (constrain .eq. 1) then
           call sample_momentum(p0, mass, beta, Natoms,
Nbeads)
-           p00(:,:,:) = p0(:,:,:)
-           call constrain_momentum_to_dividing_surface(p00,
dxi, Natoms, Nbeads)
-           p = p + p0 - p00
+           ! DAS p00(:,:,:) = p0(:,:,:)
+           ! DAS call
constrain_momentum_to_dividing_surface(p00, dxi, Natoms,
Nbeads)
+           ! DAS p = p + p0 - p00
        end if

        ! Switch to mass-scaled coordinates when storing
momenta in gle_p
@@ -1219,7 +1276,7 @@
        end do

        ! If desired, constrain the momentum to the dividing
surface
-       if (constrain .eq. 1) call
constrain_momentum_to_dividing_surface(p, dxi, Natoms,
Nbeads)
+       ! DAS if (constrain .eq. 1) call
constrain_momentum_to_dividing_surface(p, dxi, Natoms,
Nbeads)

    end subroutine gle_thermostat
```

file input.py.patch

```
--- RPMDrate-master/rpmdrate/input.py           2024-05-19
17:49:08.890936000 -0500
+++ ../rpmdBD316aa/rpmdrate/input.py            2024-05-19
18:44:55.540161782 -0500
@@ -87,7 +87,7 @@
    global jobList
    jobList.append(['PMF', (windows, xi_min, xi_max, bins)])

-def computeRecrossingFactor(dt, equilibrationTime,
childTrajectories, childSamplingTime, childrenPerSampling,
childEvolutionTime, xi_current=None,
saveParentTrajectory=False, saveChildTrajectories=False):
+def computeRecrossingFactor(dt, equilibrationTime,
childTrajectories, childSamplingTime, childrenPerSampling,
childEvolutionTime, xi_current=None,
saveParentTrajectory=True, saveChildTrajectories=True):
    global jobList
    jobList.append(['recrossing', (dt, equilibrationTime,
childTrajectories, childSamplingTime, childrenPerSampling,
childEvolutionTime, xi_current, saveParentTrajectory,
saveChildTrajectories)])

@@ -96,9 +96,9 @@
    jobList.append(['rate', tuple()])

 getPotential = None
-def potential(q):
+def potential(q,numAtoms,numBeads,DASflag,t):
    global getPotential
-   return getPotential(q)
+   return getPotential(q,numAtoms,numBeads,DASflag,t)


##################################################
#############################
```

file rpmdrate.py.patch

```
--- RPMDrate-master/rpmdrate.py         2024-05-19
17:49:08.997899000 -0500
+++ ../rpmdBD316aa/rpmdrate.py          2024-05-17
17:53:40.874631000 -0500
@@ -60,6 +60,7 @@
    parser.add_argument('T', metavar='TEMP', type=float,
nargs=1, help='the temperature in K')
    parser.add_argument('Nbeads', metavar='BEADS',
type=int, nargs=1, help='the number of beads')
    parser.add_argument('-p', '--processes', metavar='PROC',
type=int, nargs=1, default=[1], help='the number of processors
to use')
```

```
+   print("DAS in parsing command line arguments")

    # Options for controlling the amount of information printed to the console
    # By default a moderate level of information is printed; you can either
@@ -90,6 +91,7 @@
    logging.addLevelName(logging.INFO, '')
    logging.addLevelName(logging.DEBUG, '')
    logging.addLevelName(0, '')
+   print("DAS in initiaze log")

    # Create formatter and add to handlers
    formatter = logging.Formatter('%(levelname)s%(message)s')
@@ -157,6 +159,7 @@

    # Determine the output directory
    outputDirectory = os.path.dirname(os.path.abspath(args.file[0]))
+   print("DAS output directory",outputDirectory)

    # Initialize the logging system (both to the console and to a file in the
    # output directory)
@@ -169,20 +172,25 @@
    from rpmdrate.input import loadInputFile
    system, jobList = loadInputFile(args.file[0], args.T[0], args.Nbeads[0], args.processes[0])
    logging.info('')
+   print("DAS args.file",args.file[0], "args.T",args.T[0], "args.Nbeads",args.Nbeads[0], "args.processes",args.processes[0])

    # Run the requested jobs
    for job, params in jobList:
        if job == 'configurations':
            dt, evolutionTime, xi_list, kforce = params
+           print("DAS configurations dt, evolutionTime, xi_list, kforce",dt, evolutionTime, xi_list, kforce)
            system.generateUmbrellaConfigurations(dt, evolutionTime, xi_list, kforce)
        elif job == 'umbrella':
            dt, windows, saveTrajectories = params
+           print("DAS umbrella dt, windows, saveTrajectories",dt, windows, saveTrajectories)
            system.conductUmbrellaSampling(dt, windows, saveTrajectories)
        elif job == 'PMF':
            windows, xi_min, xi_max, bins = params
+           print("DAS windows windows, xi_min, xi_max, bins",windows, xi_min, xi_max, bins)
            system.computePotentialOfMeanForce(windows, xi_min, xi_max, bins)
        elif job == 'recrossing':
            dt, equilibrationTime, childTrajectories, childSamplingTime, childrenPerSampling, childEvolutionTime, xi_current, saveParentTrajectory, saveChildTrajectories = params
+           print("DAS dt, equilibrationTime, childTrajectories, childSamplingTime, childrenPerSampling, childEvolutionTime, xi_current, saveParentTrajectory, saveChildTrajectories", dt, equilibrationTime, childTrajectories, childSamplingTime, childrenPerSampling, childEvolutionTime, xi_current, saveParentTrajectory, saveChildTrajectories)
            system.computeRecrossingFactor(dt, equilibrationTime, childTrajectories, childSamplingTime, childrenPerSampling, childEvolutionTime, xi_current, saveParentTrajectory, saveChildTrajectories)
        elif job == 'rate':
            system.computeRateCoefficient()
```

<u>file main.py.patch</u>

```
--- RPMDrate-master/rpmdrate/main.py2024-05-19 17:49:08.912117000 -0500
+++ ../rpmdBD316aa/rpmdrate/main.py          2024-05-17 17:53:40.852854000 -0500
@@ -58,6 +58,8 @@
    Run an individual umbrella integration trajectory, returning the sum of the
    first and second moments of the reaction coordinate at each time step.
    """
+   #DAS For our purposes, runUmbrellaTrajectory is not useful and could be killed.
+   print("DAS in main.py in runUmbrellaTrajectory")
    if xi_range is None: xi_range = 0.0
    rpmd.activate()
    steps = 0
@@ -80,6 +82,8 @@
    sample in the positive and negative directions of the initial sampled
    momenta.
    """
+   #DAS
+   print("DAS in main.py in runRecrossingTrajectory")
    rpmd.activate()
    result1 = 1; result2 = 1
    while result1 != 0 or result2 != 0:
@@ -89,6 +93,10 @@
        q1 = q.copy('F')
        kappa_num1 = numpy.zeros(evolutionSteps, order='F')
        kappa_denom1 = numpy.array(0.0, order='F')
+       #DAS This is the unconstrained trajectories after prior equilibration steps
+       print('DAS in main.py in runRecrossingTrajectory, part 1, looking at passed parameters')
+ #print('t1,p1,q1,xi_current,rpmd.potential',t1,p1,q1,xi_current, rpmd.potential)
+       #print('saveTrajectory, kappa_num1, kappa_denom1',saveTrajectory, kappa_num1, kappa_denom1)
        result1 = system.recrossing_trajectory(t1, p1, q1, xi_current, rpmd.potential, saveTrajectory, kappa_num1, kappa_denom1)
        if result1 != 0: continue

@@ -98,6 +106,8 @@
        q2 = q.copy('F')
        kappa_num2 = numpy.zeros(evolutionSteps, order='F')
```

```
        kappa_denom2 = numpy.array(0.0, order='F')
+        #DAS This looks to be only the unconstrained
trajectories after prior equilibration steps
+        print('DAS in main.py in runRecrossingTrajectory, part
2, looking at passed parameters')
        result2 = system.recrossing_trajectory(t2, p2, q2,
xi_current, rpmd.potential, saveTrajectory, kappa_num2,
kappa_denom2)
        if result2 != 0: continue

@@ -392,12 +402,16 @@
        # First start at xi = 1 and move in the xi > 1 direction,
using the
        # result of the previous xi as the initial position for the
next xi
        q_initial = numpy.zeros((3,self.Natoms,Nxi), order='F')
+        print("DAS here in main.py in generating umbrella
configs, q_initial, start, Nxi",q_initial, start, Nxi)
        for l in range(start, Nxi):
            xi_current = xi_list[l]

            # Equilibrate in this window
            logging.info('Generating configuration at xi = {0:.4f}
for {1:g} ps...'.format(xi_current, evolutionSteps * self.dt *
2.418884326505e-5))
            p = self.sampleMomentum(Nbeads=Nbeads)
+            #DAS need to understand equilibration and its
constraints
+            print("DAS need to understand equilibration and its
constraints")
+            print("0, p, q, evolutionSteps, xi_current,
self.potential, kforce[l], False, False",0, p, q, evolutionSteps,
xi_current, self.potential, kforce[l], False, False)
            result = system.equilibrate(0, p, q, evolutionSteps,
xi_current, self.potential, kforce[l], False, False)
            logging.info('Finished generating configuration at xi =
{0:.4f}.'.format(xi_current))
            q_initial[:,:,l] = q[:,:,0]
@@ -627,6 +641,8 @@
        using the given number of bins. This requires that you
have previously
        used umbrella sampling to determine the mean and
variance in each bin.
        """
+        # DAS
+        print("DAS in main.py in
computePotentialOfMeanForce")
        # Set up output files and directory
        workingDirectory = self.createWorkingDirectory()
        potentialFilename = os.path.join(workingDirectory,
'potential_of_mean_force.dat')
@@ -716,8 +732,8 @@
                              childrenPerSampling,
                              childEvolutionTime,
                              xi_current=None,
-                             saveParentTrajectory=False,
-                             saveChildTrajectories=False):
+                              saveParentTrajectory=True,
+                              saveChildTrajectories=True):
        """
        Return the recrossing factor for the RPMD system. A
constrained RPMD
        simulation is initiated in the presence of a thermostat to
generate a
@@ -742,6 +758,7 @@
        This is off by default because it is very slow.
        """

+        print("DAS in main.py in computeRecrossingFactor")
        # If xi_current not specified, use the maximum of the
potential of mean force
        if xi_current is None:
            if self.potentialOfMeanForce is None:
@@ -766,12 +783,14 @@
        processes = self.processes

        # Load the geometry from the closest umbrella
configuration
-        for xi, q in self.umbrellaConfigurations:
-            if xi >= xi_current:
-                geometry = q
-                break
-        else:
-            geometry = self.transitionStates[0].geometry
+        # DAS we want to just take the geometry from input.py,
not worrying about a useless (for us) set of umbrella
configurations
+        #for xi, q in self.umbrellaConfigurations:
+        #    if xi >= xi_current:
+        #        geometry = q
+        #        break
+        #else:
+        #    geometry = self.transitionStates[0].geometry
+        geometry = self.transitionStates[0].geometry

        # Initialize parameters used to compute recrossing factor
        kappa_num = numpy.zeros(childEvolutionSteps,
order='F')
```

<u>file surface.py.patch</u>

```
--- RPMDrate-master/rpmdrate/surface.py          2024-05-19
17:49:08.888358000 -0500
+++ ../rpmdBD316aa/rpmdrate/surface.py          2024-05-17
17:53:40.850444000 -0500
@@ -67,12 +67,18 @@
    def __init__(self, geometry, formingBonds,
breakingBonds):

        self.geometry =
numpy.array(quantity.convertLength(geometry, "bohr")).T
+        #DAS
+        print("self.geometry",self.geometry)

        self.formingBonds = numpy.array(formingBonds,
numpy.int)
+        #DAS
+        print("self.formingBonds",self.formingBonds)
        self.breakingBonds = numpy.array(breakingBonds,
numpy.int)

        Nforming_bonds = self.formingBonds.shape[0]
```

```
        Nbreaking_bonds = self.breakingBonds.shape[0]
+        #DAS
+        print("self.breakingBonds, Nforming_bonds, Nbreaking_bonds",self.breakingBonds, Nforming_bonds, Nbreaking_bonds)

        self.formingBondLengths = numpy.empty(Nforming_bonds)
        self.breakingBondLengths = numpy.empty(Nbreaking_bonds)
@@ -120,6 +126,8 @@
        `position`.
        """
        self.activate()
+        print("transition state position",position)
+        print("transition_state.value(position)",transition_state.value(position))
        return transition_state.value(position)

    def gradient(self, position):
@@ -128,6 +136,7 @@
        `position`.
        """
        self.activate()
+        print("transition_state.gradient(position)",transition_state.gradient(position))
        return transition_state.gradient(position)

    def hessian(self, position):
@@ -136,6 +145,7 @@
        `position`.
        """
        self.activate()
+        print("transition_state.hessian(position)",transition_state.hessian(position))
        return transition_state.hessian(position)

####################################################################################
@@ -204,6 +214,8 @@
        `position`.
        """
        self.activate()
+        print("reactants position",position)
+        print("reactants.value(position)",reactants.value(position))
        return reactants.value(position)

    def gradient(self, position):
@@ -212,6 +224,7 @@
        `position`.
        """
        self.activate()
+        print("reactants.gradient(position)",reactants.gradient(position))
        return reactants.gradient(position)

    def hessian(self, position):
@@ -220,4 +233,5 @@
        `position`.
        """
        self.activate()
+        print("reactants.hessian(position)",reactants.hessian(position))
        return reactants.hessian(position)
```

gforce.py

The python module gforce.py sets up parallel Gaussian calculations, extracts the forces, adds in constraints, and reports back to RPMDrate. It also analyzed the ongoing trajectories, makes output files giving each trajectory along with a summary in *dynfollowfile*. Finally, it reports back to RPMDrate if a trajectory is identified as complete so that a new one can be started.

```
import numpy as np
import pdb
import subprocess
import os
from datetime import datetime
def get_potential(q,numAtoms,numBeads,DASflag=None,trajtime=None):
   info = 0
   trajtime = str(round(trajtime*0.02418884326505,1))
   currentDateAndTime = datetime.now()
   currentTime = currentDateAndTime.strftime("%b-%d-%Y %H:%M:%S")
   q = np.array(q)
   q = 0.529177 * q  # q is in bohr, must convert to Angstroms for use by Gaussian

   # at its start, the program must be supplied with a text file isomernumber
   # that contains a number that will be incremented as trajectory legs finish
   isomer = doIsomernumber(DASflag,trajtime)
```

```
    # Constraints are generally applied during the equilibration
phase of calculations.
    # The format of each constraint is
[(atomnumber,atomnumber,desired distance),(constant
force,harmonic force constant,cubic force constant,quartic)]
    trajStartPointA =
[[(3,4,3.388),[0,0.5,0,0]],[(2,4,3.807),[0,0.5,0,0]]]
    noConstraint = []
    if ((DASflag == 7) or (DASflag == 40)):
        forcelist = noConstraint
    if (DASflag == 11):
        forcelist = trajStartPointA
    if (DASflag == 6):
        forcelist = noConstraint

    #initialize some parameters
    elements = ['C','C','C','B','H','H','H','H','H','H','H','H','H']
    nBeads =  np.shape(q)[-1]
    beads = np.transpose(q,axes=(2,1,0))


    # *** main process of setting up and running each Gaussian
calculation for each bead separately and simultaneously ***
    total_procs = 32    # varied with system availability of
processors
    threads = int(np.floor(total_procs/nBeads))  # the number of
processors given to each Gaussian calculation
    # we round down to avoid overassigning procs

    job_header = '%nproc={}\n%chk=bead{}.chk\n# B3LYP/6-
31g* force nosymm
scf=(xqc,maxconven=155,fulllinear,nosym)\nguess=tcheck\n\
nrpmd force calc\n\n0 1\n'
    job_command = ' g16 < bead{}.com' #we'll run this for
output
    # Before RPMDrate starts, the job submission file should
copy an old checkpoint file to bead0.chk, bead1.chk...etc
    dirlog = subprocess.check_output( 'echo
$TMPDIR',shell=True ).strip() #modify for local system.
"$TMPDIR" is our Gaussian working directory
    origindir = os.getcwd()
    if dirlog:
        os.chdir(dirlog)

    #make coordinate blocks from the raw arrays
    text_beads = []
    processes = []

    for bead_num,xyz in enumerate(beads):
      coords = ''

      #get the coordinate grid
      for number, line in enumerate(xyz):
        coords += elements[number]+' '+' '.join(str(coord) for
coord in line)+'\n'
      job = job_header.format(threads,bead_num)+coords+'\n'
      #make a file
      f=open("bead{}.com".format(bead_num),'w')
      f.write(job)
      f.close()
      #create a job process
      processes +=
[ subprocess.Popen(job_command.format(bead_num),
shell=True, stdout=subprocess.PIPE) ]

    #wait for the processes to finish
    for proc in processes:
        proc.wait()
    log = ''.join( [ process.stdout.read() for process in
processes ] )

    os.chdir(origindir)
    # code to be uncommented if one wants to look at the
Gaussian output files
    #f=open('g16.log','r')
    #log = f.read()
    #f.close()
    #print(log)    # allows printing of the gaussian file

    #process the output data
    energies = []
    forces = []
    jobs = log.split('Elapsed')[:-1] #discard the last few words
after the last 'Elapsed'
    for job in jobs:
      output = engrad(job)
      energies += [output[0]] #we want an array of energies, so
we ad brackets, else we get an array of characters
      forces += [output[1]]

    # add the forces for the constraints for reporting back to
RPMDrate
    # the routine says harmonic but the constraints are not
necessarily just harmonic
    if forcelist is not None:
        for force in forcelist:
            for index, bead in enumerate(beads):
                forces[index] += addharmonic(bead,force)


    # *** starting data analysis and reporting ***
    #make a trajectory file that can be read directly
    avgcoords = ''
    for number, line in enumerate(np.mean(beads,axis=0)):
      avgcoords += elements[number]+' '+' '.join(str(coord) for
coord in line)+'\n'
    text2 =
str(len(elements))+"\n"+str(627.509*(np.mean(energies)+144.
523976652))+" "+currentTime+"\n"+avgcoords
    if (DASflag == 6):
        ft=open("trajrecrossing",'a')
        ft.write(text2)
        ft.close()
    if (DASflag == 11):
        ft=open("trajequil",'a')
        ft.write(text2)
        ft.close()
    if ((DASflag == 7) or (DASflag == 40)):
        ft=open("trajother",'a')
        ft.write(text2)
        ft.close()

    # report desired distances to dynfollowfile, check if done
```

```
    # this section would vary from system to system
    dynftext = trajtime + " ps "
    C3B = atomdistance(np.mean(beads,axis=0),3,4)
    C2B = atomdistance(np.mean(beads,axis=0),2,4)
    C3H10 = atomdistance(np.mean(beads,axis=0),3,10)
    C3H11 = atomdistance(np.mean(beads,axis=0),3,11)
    C3H12 = atomdistance(np.mean(beads,axis=0),3,12)
    C2H10 = atomdistance(np.mean(beads,axis=0),2,10)
    C2H11 = atomdistance(np.mean(beads,axis=0),2,11)
    C2H12 = atomdistance(np.mean(beads,axis=0),2,12)
    C3Hmin = min(C3H10,C3H11,C3H12); C2Hmin =
min(C2H10,C2H11,C2H12)

    if (DASflag == 6):
       if ((C3B < 1.6) and (C2Hmin < 1.2)): info = -2
       if ((C2B < 1.6) and (C3Hmin < 1.2)): info = -3
       if ((C3B > 3.5) and (C2B > 3.9)): info = -4
       if (trajtime == "2999.0"): info = -5
    C3B = '{:.4}'.format(C3B); C2B = '{:.4}'.format(C2B);
C3Hmin = '{:.4}'.format(C3Hmin); C2Hmin =
'{:.4}'.format(C2Hmin)
    dynftext = dynftext + " C3B " + C3B + "  C2B " + C2B + "
C3Hmin " + C3Hmin + " C2Hmin " + C2Hmin
    if (info == -2): dynftext = dynftext + " XXXX anti-Mark
product"
    if (info == -3): dynftext = dynftext + " XXXX markovnikov
product"
    if (info == -4): dynftext = dynftext + " XXXX returned SM"
    if (info == -5): dynftext = dynftext + " XXXX Too many
points"
    dynftext = dynftext +" "+currentTime+" DASflag
"+str(DASflag)+" isomernumber "+str(isomer)+" \n"
    fdynf=open("dynfollowfile",'a')
    fdynf.write(dynftext)
    fdynf.close()

    return energies,-np.transpose(forces,axes=(2,1,0)),info



def engrad(block):
    #block is a gaussian output in string format, as is generated
by file.read()
    lines = iter(block.split('\n'))
    force = []
    for line in lines:
       if 'Done' in line: energy = float(line.split()[4])
       if 'Hartrees/Bohr' in line:
          lines.next() #this is the decorative line,
          lines.next() # we are skipping it
          nuline = lines.next()
          while len(nuline.split()) == 5:
             force += [[float(number) for number in nuline.split()[-
3:]]]
             nuline = lines.next()
          return energy, force  # this is the energy of the vts

def doIsomernumber(DASflag,trajtime):
    with open("isomernumber","r") as f:
       isomer = f.readline().strip()
       childflag = str(int(isomer)+1)
    if (DASflag == 40):
       if (int(isomer) >= 0):
          newshellcommand = "mv trajrecrossing
trajrecrossing"+isomer+"; mv equil_cent_trj
equil_cent_trj"+isomer+"; mv child.xyz
child.xyz"+childflag+"; mv child_centroid.xyz
child_centroid.xyz"+childflag
          movinglog =
subprocess.check_output( newshellcommand,shell=True )
          print
"newshellcommand",newshellcommand,"isomer",isomer,"chil
dflag",childflag
       with open("isomernumber","w") as f:
          isomer = int(isomer) + 1
          f.write(str(isomer)+"\n")
    return isomer

def atomdistance(geom,atomnumber1,atomnumber2):
    atom1 = np.array(geom[atomnumber1-1])  # the array starts
at 0, so subtract 1 here
    atom2 = np.array(geom[atomnumber2-1])
    rhat = atom2-atom1
    r = np.linalg.norm(rhat)
    return r

def addharmonic(geom, forces):  # bad name, since forces may
not be just harmonic, though most often they are
    #geom is an Nx3 block of coordinates for some N atoms
    #forces is an array of two lists: (also not the same forces as
in getpotential)
    #the first contains exactly three numbers corresponding to
the atom numbers and the "equilibrium" distance
    #the force should be applied between. NOT ZERO
INDEXED
    #the second contains a number coefficient for the mth term
in a polynomial
    #eg [ (1,2,1.5), (-4 , 5, 6)] find the distance between the first
two atoms, subtract 1.5 from it to get r
    #and applies the force 6r^2+5r-4 along the internuclear axis
    #this returns an array of the same dimensions as geom, with
just the requested forces as nonzero terms
    #sign convention should be positive numbers result in

    #get coordinates
    atom1 = np.array(geom[forces[0][0]-1]) #arrays, of course,
*are* zero-indexed
    atom2 = np.array(geom[forces[0][1]-1])
    rhat = atom2-atom1
    r=np.linalg.norm(rhat)
    rhat=rhat/r
    r += -forces[0][2] #adjust r by the equilibrium distance

    #generate force
    power= 1
    force = np.zeros(3)
    for coeff in forces[1]:
       force += coeff*power*rhat
       power = power*r

    #now make the force array
    output= np.zeros(np.shape(geom))
    output[forces[0][0]-1] += force
    output[forces[0][1]-1] += -force #equal and opposite
```

```
    return output

testq= \
        [[[ 0.000000 ],
        [ -0.000000],
        [0.000000 ],
        [-0.000000],
        [0.000000 ],
        [-0.000000],
        [0.917595 ],
        [-0.917595],
        [-0.917595],
        [0.917595 ]],

        [[0.589215   ],
        [ -0.589215 ],
        [0.973842   ],
        [ -0.973842 ],
        [0.684154   ],
        [ -0.684154 ],
        [1.234787   ],
        [ 1.234787  ],
        [ -1.234787 ],
        [-1.234787  ]],

        [[  1.418891],
        [   1.418891],
        [ 0.324514],
        [ 0.324514],
        [ -1.241109],
        [ -1.241109],
        [ -1.405049],
        [ -1.405049],
        [ -1.405049],
        [ -1.405049]]]
testq = np.array(testq)/0.529177

if __name__ == '__main__':
    block =
get_potential(testq,[[(2,3,1.1),[0,.1,0]],[(1,4,1.1),[0,.1,0,0]]])
#for debugging
    print("output is:\n"+str(block))
```

input.py

The *input.py* file is the normal input for RPMDrate, simplified here because no umbrella sampling was performed. The geometry given is the starting geometry for equilibration. The deuteroboration trajectories differed only by replacing H with D for atoms 10, 11, and 12.

```
#!/usr/bin/env python
# encoding: utf-8

from gforce import get_potential

################################################################################

label = 'BH3 + propene -> CH3CH2CH2BH2'

reactants(
    atoms = ['C', 'C', 'C', 'B', 'H', 'H', 'H', 'H', 'H', 'H', 'H', 'H', 'H'],
    reactant1Atoms = [1,2,3,4],
    reactant2Atoms = [5,6,7,8,9,10,11,12,13],
    Rinf = (30 * 0.52918,"angstrom"),
)

transitionState(
    geometry = (
        [[ 1.804733,   -0.709183,    0.170106],
         [ 0.839020,    0.252441,   -0.460550],
         [ 0.082688,    1.133617,    0.201331],
         [ -2.851904,  -0.550134,    0.016632],
         [ 2.827028,   -0.530462,   -0.189709],
         [ 1.557781,   -1.746893,   -0.090215],
         [ 0.776538,    0.220380,   -1.549202],
         [ 0.116733,    1.213306,    1.286395],
         [ -0.578890,   1.822263,   -0.316660],
         [ -3.258990,   0.481010,   -0.427460],
         [ -2.796535,  -0.710540,    1.198415],
         [ -2.549386,  -1.438474,   -0.721537],
         [ 1.806593,   -0.621171,    1.261489]],
```

```
        "angstrom"),
    formingBonds = [(4,6),(3,5)], # not used
    breakingBonds = [(1,4),(2,3)], # not used
)


thermostat('Andersen')


computeRecrossingFactor(
    dt = (0.0005,"ps"),
    equilibrationTime = (1.0,"ps"),
    childTrajectories = 1000,
    childSamplingTime = (0.050,"ps"),
    childrenPerSampling = 2,
    childEvolutionTime = (3.00,"ps"),
    xi_current = 0.86,
)
```

A Sample Slurm Script

To aid researchers who aim to reproduce the work here or apply the modified RPMDrate program to other problems, we include a sample SLURM batch submission script. This is of course system-dependent and will need to be modified for the local environment.

file_rpmd

```
#!/bin/bash
#
#SBATCH -J rpmdBH316v -e tempeorpmdBH316v -o tempeorpmdBH316v
#SBATCH -t 1:00:00 -n 64 --mem=240G
#SBATCH --ntasks-per-node=64 #always specify 1 node for gaussian jobs

cd $TMPDIR
echo
echo working directory
pwd
echo
echo files in directory
ls
echo

export g16root=/sw/restricted/lms/sw/Gaussian/g16_C01
. $g16root/g16/bsd/g16.profile

echo -P- 64 > Default.Route
echo -M- 5GB >> Default.Route

origdir=$SLURM_SUBMIT_DIR
cd $origdir
cp BH3.chk $TMPDIR/bead0.chk
# BH3.chk is a Gaussian 16 checkpoint file for a calculation on VTS in the main text
cp BH3.chk $TMPDIR/bead1.chk
cp BH3.chk $TMPDIR/bead2.chk
cp BH3.chk $TMPDIR/bead3.chk
cp BH3.chk $TMPDIR/bead4.chk
cp BH3.chk $TMPDIR/bead5.chk
cp BH3.chk $TMPDIR/bead6.chk
cp BH3.chk $TMPDIR/bead7.chk
cp BH3.chk $TMPDIR/bead8.chk
cp BH3.chk $TMPDIR/bead9.chk
cp BH3.chk $TMPDIR/bead10.chk
```

```
cp BH3.chk $TMPDIR/bead11.chk
cp BH3.chk $TMPDIR/bead12.chk
cp BH3.chk $TMPDIR/bead13.chk
cp BH3.chk $TMPDIR/bead14.chk
cp BH3.chk $TMPDIR/bead15.chk

echo "0" > isomernumber

module purge
module load Miniconda3/23.5.2-0 intel-compilers/2023.1.0 GCC/12.3.0 OpenMPI/4.1.5 FFTW.MPI/3.3.10
conda list
__conda_setup="$('/sw/eb/sw/Miniconda3/23.5.2-0/bin/conda' 'shell.bash' 'hook' 2> /dev/null)"
if [ $? -eq 0 ]; then
   eval "$__conda_setup"
else
   if [ -f "/sw/eb/sw/Miniconda3/23.5.2-0/etc/profile.d/conda.sh" ]; then
      . "/sw/eb/sw/Miniconda3/23.5.2-0/etc/profile.d/conda.sh"
   else
      export PATH="/sw/eb/sw/Miniconda3/23.5.2-0/bin:$PATH"
   fi
fi
unset __conda_setup
export PATH=$PATH:/scratch/user/d-singleton/.conda/envs/27rpmd
source activate 27rpmd
python --version

GFORTRAN_UNBUFFERED_ALL='y'
export GFORTRAN_UNBUFFERED_ALL

hostname >> $origdir/docslog
date >> $origdir/docslog
echo "beginning time rpmd5-16D1b" >> $origdir/docslog
cat BH3/input.py >> $origdir/docslog
cat BH3/gforce.py >> $origdir/docslog

mv dynfollowfile bak.dynfollowfile
mv BH3/pyrate.log BH3/temp.log

python -u rpmdrate.py BH3/input.py 298.15 16

cd $TMPDIR
echo "files in TMPDIR"
ls
echo
date >> $origdir/tempeorpmdBH316v
echo "ending time rpmdBH316v" >> $origdir/docslog
date >> $origdir/docslog

echo "" >> $origdir/docslog
echo working directory
pwd
echo
echo files in directory
ls -al
echo

exit
```

Helper and Data-Analysis Programs

script file progcount

```
# A script program to analyze and organize the results from a series of directories containing RPMD calculations.
echo propene 16
grep XXX savedBH316/*/dynf* rpmdBH316*/dynf* | sed 's/:/ /' | awk '{
    countlines++}; i
    /returned/ {countret++; rettime[countret]=$2}
    /mark/ {countmark++; marktime[countmark]=$2; totmarktime=totmarktime+$2};
    /Mark/ {countMark++; Marktime[countMark]=$2; totMarktime=totMarktime+$2}
    /Too/ {counttoo++};
  END {print counttoo,"with too many points";
    for (i=1;i<=countmark;i++) {print marktime[i]}
    print ""
    n = 1 + int(0.5*asort(Marktime)); m = 1 + int(0.5*asort(marktime)); o = 1 + int(0.5*asort(rettime))
    print countMark,"anti-Markovnikov, with an average time of",totMarktime/countMark,"and a median time of",Marktime[n]
    for (i=1;i<=countMark;i++) {printf(" %i",Marktime[i])}
    print ""
    print countmark,"markovnikov product, with an average time of",totmarktime/countmark,"and a median time
of",marktime[m]
    for (i=1;i<=countmark;i++) {printf(" %i",marktime[i])}
    print ""
    print countret,"returned to starting material"
    print countlines,"total completed trajectory legs"
    print "anti-Markovnikov   markovnikov   Too many point   Returned to SM   percentages"

printf("     %.1f            %.1f          %.1f            %.1f\n",100*countMark/countlines,100*countmark/countlines,100*counttoo/cou
ntlines,100*countret/countlines)
    interesting=countlines-countret
    print "anti-Markovnikov   markovnikov   Too many point "
    printf("     %.1f            %.1f          %.1f
\n",100*countMark/interesting,100*countmark/interesting,100*counttoo/interesting)}'
echo ""
```

awk program proggmmx

```
# This is an awk program that combines child.xyz and child_centroid.xyz trajectory files from RPMD runs into a single file
# in the gmmx format of GaussView 6. This provides a way to graphically depict the RPMD trajectories, as used in the
# main text figures.  The program also keeps track of the average distance of trajectories beads from the centroid.
BEGIN {
# ask for a specific file number by feeding this way: awk -v file=theNumberYouWant somesimpletextfilethatisignored
if (file<1) {
  print "Usage: awk -v file=theNumberYouWant -f proggmmx somesimpletextfilethatisignored"
  print "where theNumberYouWant is the number at the end of your choice of child and child_centroid xyz files"
  print "and somesimpletextfilethatisignored is really just any file because awk requires you to have an input file"
  exit
  }
childfile="child.xyz"file
centroidfile="child_centroid.xyz"file
distancefile="distances"file

CHmin=1.05
CHmax=3.05
CHbinsize=0.1
CHbins=int((CHmax-CHmin)/CHbinsize)
for (i=0;i<=CHbins;i++) {
  H10sprd[i] = 0; H10count[i] = 0
  H11sprd[i] = 0; H11count[i] = 0
  H12sprd[i] = 0; H12count[i] = 0
  }
```

```
endstring="0  0  0  0  0  0  0  0  0  0  0  0"
zerostring="0 0 0 0 0 0 0 0 0 0"

for (j=1;j<=8000;j++) {
  connections = 8 # may be added to later
  for (i=1;i<=20;i++) {
    addcon[i]=""
    }
  getline < centroidfile
  if (j==1) centroidatoms = $1
  finishcheck = getline < childfile
  if (finishcheck == 0) exit
  if (j>1) print "$$$$"
  if (j==1) childatoms = $1
  totalatoms = centroidatoms + childatoms
  beads = childatoms/centroidatoms

  getline < centroidfile
  getline < childfile
  for (i=1;i<=centroidatoms;i++) {
    getline < centroidfile
    A[i] = $2; B[i] = $3; C[i] = $4; element[i]=$1
    }
  if ((Distance(4,3)<2.2) && (Distance(4,3)-Distance(4,2)<.2)) {connections++; addcon[1]="  3  4  1  0  0  0  0"}
  if ((Distance(4,2)<2.2) && (Distance(4,2)-Distance(4,3)<.2)) {connections++; addcon[2]="  2  4  1  0  0  0  0"}
  if (Distance(10,3)<1.7) {connections++; addcon[3]="  3 10  1  0  0  0  0"}
  if (Distance(11,3)<1.7) {connections++; addcon[4]="  3 11  1  0  0  0  0"}
  if (Distance(12,3)<1.7) {connections++; addcon[5]="  3 12  1  0  0  0  0"}
  if (Distance(10,2)<1.7) {connections++; addcon[6]="  2 10  1  0  0  0  0"}
  if (Distance(11,2)<1.7) {connections++; addcon[7]="  2 11  1  0  0  0  0"}
  if (Distance(12,2)<1.7) {connections++; addcon[8]="  2 12  1  0  0  0  0"}
  if (Distance(10,4)<1.3) {connections++; addcon[9]="  4 10  1  0  0  0  0"}
  if (Distance(11,4)<1.3) {connections++; addcon[10]="  4 11  1  0  0  0  0"}
  if (Distance(12,4)<1.3) {connections++; addcon[11]="  4 12  1  0  0  0  0"}

  print ""
  print "  "j
  printf("%i ",j) > distancefile
  shortC2H = Distance(2,10); if (Distance(2,11)<shortC2H) shortC2H=Distance(2,11); if (Distance(2,12)<shortC2H)
shortC2H=Distance(2,12)
  shortC3H = Distance(3,10); if (Distance(3,11)<shortC3H) shortC3H=Distance(3,11); if (Distance(3,12)<shortC3H)
shortC3H=Distance(3,12)
  whichH = 10; if (Distance(3,11)==shortC3H) whichH = 11; if (Distance(3,12)==shortC3H) whichH = 12
  print
"C3B",Distance(4,3),"C2B",Distance(4,2),"shortC2H",shortC2H,"shortC3H",shortC3H,"H10spd",Beadspread(10),"H11spd",Bea
dspread(11),"H12spd",Beadspread(12) > distancefile
  a=int((shortC3H-CHmin)/CHbinsize)+1
  H10sprd[a] = H10sprd[a] + Beadspread(10); H10count[a]++
  H11sprd[a] = H11sprd[a] + Beadspread(11); H11count[a]++
  H12sprd[a] = H12sprd[a] + Beadspread(12); H12count[a]++

  print ""
  if (totalatoms<100) printf(" %i %i %s",totalatoms,connections,zerostring)
  if (totalatoms>100) printf("%i %i %s",totalatoms,connections,zerostring)
  print ""
  for (i=1;i<=centroidatoms;i++) {
    printf("   %7.4f   %7.4f   %7.4f %s   %s",A[i],B[i],C[i],element[i],endstring)
    print ""
    }
  for (i=centroidatoms+1;i<=centroidatoms+childatoms;i++) {
    getline < childfile
    A[i] = $2; B[i] = $3; C[i] = $4; element[i]=$1
```

```
    printf("   %7.4f   %7.4f   %7.4f %s   %s",$2,$3,$4,$1,endstring)
    print ""
    }
  print "  1  2  1  0  0  0  0"
  print "  1  5  1  0  0  0  0"
  print "  1  6  1  0  0  0  0"
  print "  1 13  1  0  0  0  0"
  print "  2  3  1  0  0  0  0"
  print "  2  7  1  0  0  0  0"
  print "  3  8  1  0  0  0  0"
  print "  3  9  1  0  0  0  0"
  for (i=1;i<=20;i++) {
    if (length(addcon[i])>1) print addcon[i]
    }
  }
}
END {
print "M END"
print "file",file,"H reacting",whichH,"final distance",shortC3H > distancefile
for (i=2;i<=CHbins;i++) {
  printf("%.2f \t",1.00+i*0.1) > distancefile
  if ( H10count[i] > 0) printf("%.3f",H10sprd[i]/H10count[i]) > distancefile
  printf("\t") > distancefile
  if ( H11count[i] > 0) printf("%.3f",H11sprd[i]/H11count[i]) > distancefile
  printf("\t") > distancefile
  if ( H12count[i] > 0) printf("%.3f",H12sprd[i]/H12count[i]) > distancefile
  print "" > distancefile
  }

}

function Distance(Atom1,Atom2) {
 return sqrt((A[Atom1]-A[Atom2])^2 + (B[Atom1]-B[Atom2])^2 + (C[Atom1]-C[Atom2])^2)
}

function Beadspread(Atom) {
  print Atom,centroidatoms + beads*(Atom-1) + 1,centroidatoms + beads*(Atom-1) + beads > "diaglist"
  totaldistance = 0
  for (k = centroidatoms + beads*(Atom-1) + 1; k <= centroidatoms + beads*(Atom-1) + beads; k++) {
    totaldistance = totaldistance + Distance(Atom,k)
    }
  return totaldistance/beads
}
```

References

1. Frisch, M. J.; Trucks, G. W.; Schlegel, H. B.; Scuseria, G. E.; Robb, M. A.; Cheeseman, J. R.; Scalmani, G.; Barone, V.; Petersson, G. A.; Nakatsuji, H.; Li, X.; Caricato, M.; Marenich, A. V.; Bloino, J.; Janesko, B. G.; Gomperts, R.; Mennucci, B.; Hratchian, H. P.; Ortiz, J. V.; Izmaylov, A. F.; Sonnenberg, J. L.; Williams; Ding, F.; Lipparini, F.; Egidi, F.; Goings, J.; Peng, B.; Petrone, A.; Henderson, T.; Ranasinghe, D.; Zakrzewski, V. G.; Gao, J.; Rega, N.; Zheng, G.; Liang, W.; Hada, M.; Ehara, M.; Toyota, K.; Fukuda, R.; Hasegawa, J.; Ishida, M.; Nakajima, T.; Honda, Y.; Kitao, O.; Nakai, H.; Vreven, T.; Throssell, K.; Montgomery Jr., J. A.; Peralta, J. E.; Ogliaro, F.; Bearpark, M. J.; Heyd, J. J.; Brothers, E. N.; Kudin, K. N.; Staroverov, V. N.; Keith, T. A.; Kobayashi, R.; Normand, J.; Raghavachari, K.; Rendell, A. P.; Burant, J. C.; Iyengar, S. S.; Tomasi, J.; Cossi, M.; Millam, J. M.; Klene, M.; Adamo, C.; Cammi, R.; Ochterski, J. W.; Martin, R. L.; Morokuma, K.; Farkas, O.; Foresman, J. B.; Fox, D. J. Gaussian 16 Rev. C.01; Wallingford, CT, 2016.

2. Zheng, J.; Bao, J. L.; Zhang, S.; Corchado, J. C.; Meana-Paneda, R.; Chuang, Y. -Y.; Coitino, E. L.; Ellingson, B. A.; Truhlar, D. G. GAUSSRATE, version 2017/P2017-G09 University of Minnesota: Minneapolis, MN, 2017.

3. Zheng, J.; Bao, J. L.; Meana-Pañeda, R.; Zhang, S.; Lynch, B. J.; Corchado, J. C.; Chuang, Y.-Y.; Fast, P. L.; Hu, W.-P.; Liu, Y.-P.; Lynch, G. C.; Nguyan, K. A.; Jackels, C. F.; Fernandez Ramos, A.; Ellingson, B. A.; Melissas, V. S.; Villà, J.; Rossi, I.; Coitiño, E. L.; Pu, J.; Albu, T. V.; Ratkiewicz, A.; Steckler, R.; Garrett, B. C.; Isaacson, A. D.; and Truhlar, D. G.; Polyrate–version 2016-2A, University of Minnesota, Minneapolis, 2016.

4. For some early examples with RPMD, see: (a) Miller, T. F. Isomorphic classical molecular dynamics model for an excess electron in a supercritical fluid. *J. Chem. Phys.* **2008**, *129*, 194502. (b) Collepardo, R.; Suleimanov, Y. V.; Manolopoulos, D. E. Bimolecular reaction rates from ring polymer molecular dynamics. *J. Chem. Phys.* **2009**, *130*, 174713. (c) Perez, A.; Tuckerman, M. E.; Muser, M. H. A comparative study of the centroid and ring-polymer molecular dynamics methods for approximating quantum time correlation functions from path integrals. *J. Chem. Phys.* **2009**, *130*, 184105.
